# *De Novo* Centromere Formation and Centromeric Sequence Expansion in Wheat and its Wide Hybrids

**DOI:** 10.1371/journal.pgen.1005997

**Published:** 2016-04-25

**Authors:** Xiang Guo, Handong Su, Qinghua Shi, Shulan Fu, Jing Wang, Xiangqi Zhang, Zanmin Hu, Fangpu Han

**Affiliations:** 1 State Key Laboratory of Plant Cell and Chromosome Engineering, Institute of Genetics and Developmental Biology, Chinese Academy of Sciences, Beijing, China; 2 University of Chinese Academy of Sciences, Beijing, China; 3 State Key Laboratory of Plant Breeding and Genetics, Sichuan Agricultural University, Wenjiang, Chengdu, Sichuan, China; The University of North Carolina at Chapel Hill, UNITED STATES

## Abstract

Centromeres typically contain tandem repeat sequences, but centromere function does not necessarily depend on these sequences. We identified functional centromeres with significant quantitative changes in the centromeric retrotransposons of wheat (CRW) contents in wheat aneuploids (*Triticum aestivum*) and the offspring of wheat wide hybrids. The CRW signals were strongly reduced or essentially lost in some wheat ditelosomic lines and in the addition lines from the wide hybrids. The total loss of the CRW sequences but the presence of CENH3 in these lines suggests that the centromeres were formed *de novo*. In wheat and its wide hybrids, which carry large complex genomes or no sequenced genome, we performed CENH3-ChIP-dot-blot methods alone or in combination with CENH3-ChIP-seq and identified the ectopic genomic sequences present at the new centromeres. In adcdition, the transcription of the identified DNA sequences was remarkably increased at the new centromere, suggesting that the transcription of the corresponding sequences may be associated with *de novo* centromere formation. Stable alien chromosomes with two and three regions containing CRW sequences induced by centromere breakage were observed in the wheat-*Th*. *elongatum* hybrid derivatives, but only one was a functional centromere. In wheat-rye (*Secale cereale*) hybrids, the rye centromere-specific sequences spread along the chromosome arms and may have caused centromere expansion. Frequent and significant quantitative alterations in the centromere sequence via chromosomal rearrangement have been systematically described in wheat wide hybridizations, which may affect the retention or loss of the alien chromosomes in the hybrids. Thus, the centromere behavior in wide crosses likely has an important impact on the generation of biodiversity, which ultimately has implications for speciation.

## Author Summary

Chromosomal rearrangements during the formation of wheat aneuploids and their wide hybrids caused reduction, elimination or expansion of the centromeric retrotransposon sequences and the formation of multiple centromeres. Centromere function was not affected by centromeric sequence elimination, which was revealed by the *de novo* formation of centromeres on the rearranged chromosomes. Several retrotransposon-like elements near the former centromeres were embedded in the newly formed centromeres, and there were no obvious changes in six histone modifications between normal and new centromeres. The DNA sequences associated with the new centromeres are transcribed at a higher level after centromere formation. Chromosomes containing the neocentromeres can be stably transferred to the next generation. Chromosomes carrying two- or three-locus centromeres are unstable, which induces the formation of novel chromosomes through centromere breakage in wheat-*Th*. *elongatum* hybrid derivatives. The centromere-specific sequences on dicentric chromosomes are expanded to the chromosome arms in wheat-rye hybrids, and these sequences may function as a part of the active centromere to cause chromosome breakage in the next generation. Centromere variation and activity in wheat aneuploids and its wide hybrids may be associated with chromosome stability, rearrangements, and novel chromosome formations.

## Introduction

Centromeres, which are located at the primary constriction of the chromosome, are required for the accurate segregation of chromosomes and serve as the sites for kinetochore assembly during mitosis and meiosis. The main DNA components of the centromere are highly repetitive, such as the 171-bp α-satellite repeat in humans and 150- to 180-bp simple tandem repeats in some flowering plants [1–5]. Long-terminal repeat (LTR) retrotransposons, also known as centromeric retrotransposons (CRs), are often intermingled with tandem repeats and are enriched in plant centromeric regions [6–11]. The highly conserved function of the centromere is correlated with its epigenetic features, including the histone H3 variant CENH3 in plants (CENP-A in mammals) [12–15], phosphorylation of histone H2A at Thr-133 [16] and H3 phosphorylation at Ser-10 [17,18]. Despite the conserved centromere function, centromeric repeat sequences apparently evolved rapidly in some species under specific circumstances. This phenomenon is known as the "centromere paradox" [13].

Centromeric sequences are highly variable between different species and different chromosomes and even between the same centromeres from different ecotypes or varieties [5,11,19,20]. Most of the centromeric tandem repeats in plants, such as CentO in rice (*Oryza sativa*), CentBd in *Brachypodium distachyon*, and CentC in maize (*Zea mays*), are likely to be species-specific [4,5,21]. Several wild *Oryza* species lack CentO and instead possess genome-specific satellite repeats [22]. Similarly, little homology was found between the centromeric sequences of the potato (*Solanum tuberosum*) and its wild relative *S*. *verrucosum* [23]. Moreover, centromeres showed diversity in the repeat-less and repeat-based sequences on different chromosomes of *S*. *verrucosum* [20]. Eukaryotic centromeres carrying novel satellites may have evolved from neocentromeres that experienced insertion and/or extensive amplification of satellite repeats [20,24]. Previous studies revealed that recent segmental duplication, abundant rearrangements, and reshuffling occurred in CEN4 and CEN8 of rice and that the changes in CEN8 seemed to appear after the divergence of the *O*. *sativa* subspecies *japonica* and *indica* from a common ancestor [24,25]. An analysis of centromere retention or loss indicated that the major events during the evolution of maize from a supposed tetraploid ancestor (*Sorghum bicolor*) were chromosomal rearrangements, such as insertions and translocations, resulting in dysploidy and reduced chromosome numbers [26]. Despite the observation that substantial variations in centromeres occurred during evolution, the relationship between centromere variations and species evolution remains uncertain.

In most eukaryotes, the centromeric sequences alone are insufficient to maintain a functional centromere [27]. In humans and plants, many newly formed centromeres are devoid of typical centromeric sequences, and their formation was likely determined by epigenetic mechanisms [28–32]. Additionally, the centromere activity of dicentric chromosomes is independent of centromeric sequences. Many stable dicentric chromosomes in maize, including A-A and A-B centromeres (the centromere of the supernumerary B chromosome contains B-specific repeats), contain one active and one inactive centromere, as determined by examining the epigenetic modifications [18,33]. Furthermore, the inactive centromere recovered its activity by switching its epigenetic features under certain circumstances [34]. The essential structural and functional components for the core chromatin of centromeres include pericentromeric heterochromatin and active transcription of centromeric DNA [35–38]. The epigenetic components, rather than the DNA sequences, are essential for the establishment of centromere function. However, it remains a mystery why most functional centromeres contain highly repetitive sequences.

Allopolyploid wheat, either tetraploid or hexaploid, originates from interspecific hybridizations that trigger striking chromosomal rearrangements, genome reorganization, and chromatin remodeling in the parental genomes [39–43]. Wheat also has the capacity to hybridize with its wild relatives, which provides a broader gene source for wheat germplasm enhancement through addition, translocation, and substitution lines containing alien chromosomes [44–46]. In fact, wheat appears to prefer alien chromosomes or fragments from specific genomes [47]. However, the mechanisms regulating the stable transmission of these alien genomic sources in defined genetic backgrounds are still unclear. A previous study indicated that the size of the maize centromere was expanded in oat **(***Avena sativa*)-maize addition lines, which may be a key factor for the survival of neocentromeric chromosomes in natural populations [48]. As such, an understanding of the adaptation of centromeres to "genome shock" and their evolutionary history in the wide wheat hybrid will require additional studies.

**Fig 1:**
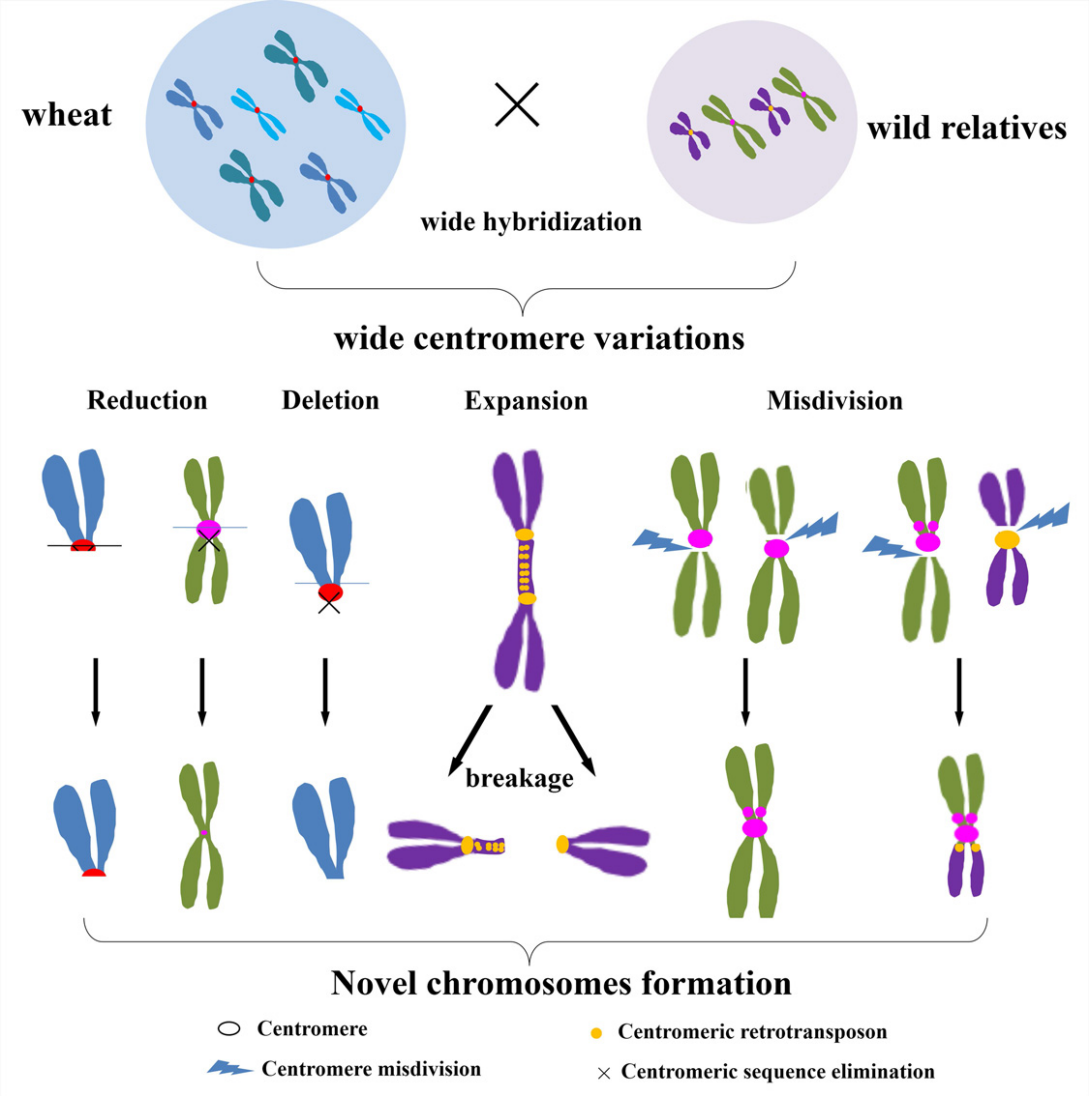
Stable and novel chromosomes induced by universal centromere variations in wheat and wide hybrids. Centromere alterations occurred in aneuploid wheat and in wheat hybrids. Reduction or even deletion of the centromeric sequences did not affect the transmission of the chromosomes or chromosome fragments to the next generation because of *de novo* centromere formation. Centromere expansion and breakage led to novel alien chromosomes in wheat and wide hybrids.

Due to their repetitive structures and low sequence conservation, it is difficult to compare centromeric sequences across different species. Complete centromeres on partial chromosomes have been sequenced in rice and maize [8,49–51]. In wheat, only partial centromere sequences have been released from published *bacterial artificial chromosome* (BAC) sequences [49,52–54]. Here, we observed that the content of classical centromeric retrotransposon sequences was reduced or apparently lost in both aneuploid wheats (4D, 1B, 5D chromosomes) and their wild relatives, such as *Th*. *intermedium* and *Th*. *elongatum*, when hybridized with wheat (Fig 1 and Table 1). With new developments in wheat genome sequencing, we first uncovered the detailed sequences in the new centromere of the 4DS chromosome in wheat aneuploids by ChIP (chromatin immunoprecipitation)-sequencing with wheat CENH3 antibodies. Additionally, for *Th*. *intermedium*, which does not have a sequenced reference genome, we developed a new ChIP-dot-blot strategy (see Methods) to identify the novel centromeric sequences in the wheat-*Th*. *intermedium* addition line TAI-14. We also detected the expansion of centromeric sequences and the formation of multiple centromeres in wheat and its wide hybrid offspring (Fig 1 and Table 1). Finally, we provide a detailed analysis of centromere variations and offer some new insights into centromere evolution in wheat and its wild relatives (Fig 1).

**Table 1:** Centromere variation and *de novo* centromere formation in wheat aneuploids and derivatives of wheat wild hybrids.

Materials	Types of centromere variation	Chromosome
Ditelosomic line 4DS	sequence deletion	4DS
Wheat-*Th*. *intermedium* addition lines	sequence deletion	alien chromosome
Wheat-*Th*. *elongatum* addition lines	sequence deletion	3E
Ditelosomic lines 1BS, 5DS, 5DL	sequence reduction	1BS,5DS,5DL
Wheat-*Th*. *elongatum* addition lines	sequence reduction	alien chromosome
N3AT3B× 8802	two-locus centromere	2E
N3DT3B	two functional centromeres	wheat and alien chromosome
N5AT5B× 8802	two functional centromeres	alien chromosome
N5BT5A× 8802	two-locus centromere	2E
N5DT5B× 8802	two-locus centromere	2E
	two functional centromeres	wheat and alien
	three functional centromeres	chromosome
	sequence reduction	alien chromosome
N6AT6B× 8802	two- and three-locus centromere	2E,5E
N6DT6A× 8802	two-locus centromere	alien chromosome
	two functional centromeres	wheat chromosome
	sequence reduction	alien chromosome
Wheat-*Th*. *poticum* addition lines	two functional centromeres	wheat chromosome
	three functional centromeres	
Wheat-*Th*. *intermedium* addition lines	two functional centromeres	wheat chromosome
Wheat-*Th*. *poticum* addition lines	two functional centromeres	wheat chromosome
Wheat and *Agropyron cristatum* addition lines	two functional centromeres	wheat chromosome
Wheat and *Hordeum vulgare* addition lines	two functional centromeres	wheat chromosome
Wheat-rye 2R addition lines	centromere expansion	2RS
Wheat-rye 6R addition lines	centromere expansion	6RS
Wheat and rye addition lines	two functional centromeres	wheat chromosome

## Results

### Reduction and elimination of centromeric sequences in wheat aneuploids and derivatives of wheat wide hybrids

The loss of canonical centromere sequences can be induced by breakage, rearrangements and radiation at plant centromeres [31,32]. Here, we observed the elimination of centromeric sequences in both wheat aneuploids and their wide hybrids.

**Fig 2:**
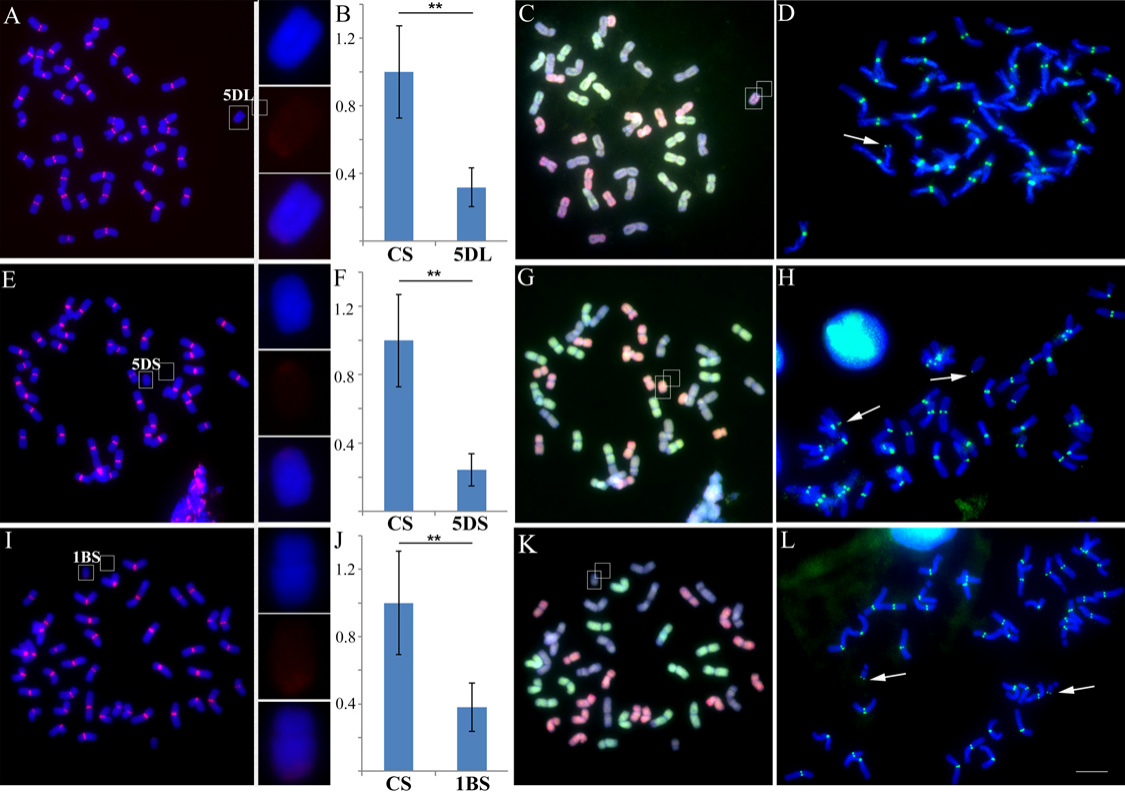
Centromeric sequence reduction in the ditelosomic lines 5DL, 5DS and 1BS. (A), (E) and (I). FISH analysis of 5DL, 5DS, and 1BS, respectively. The CRW sequences are labeled in red, and DAPI staining is labeled in blue. The insets show high-magnification images of chromosomes 5DL, 5DS and 1BS. (B), (F) and (J). Intensity of CRW fluorescence in 5DL, 5DS and 1BS, respectively, compared with the controls in Chinese Spring. The Y-axis represents the relative CRW fluorescence intensity. The error bars show the standard deviation (s.d.). The double asterisks denote significant differences at P<0.001 (two-tailed Student’s t-test). P = 3.4E-16 (B), P = 8.22E-18 (F), P = 1.97E-13 (J). (C), (G) and (J). Multi-color FISH analysis of 5DL, 5DS, and 1BS, respectively. The DNA for the wheat A genome is labeled in green, the DNA for the D genome is labeled in red, and the DNA for the B genome is used as a block. (D), (H) and (L). Immunostaining of 5DL, 5DS, and 1BS, respectively, with antibodies against CENH3 (green). The arrows indicate CENH3 on the 5DL, 5DS and 1BS chromosomes. Bar = 10 μm.

**Fig 3:**
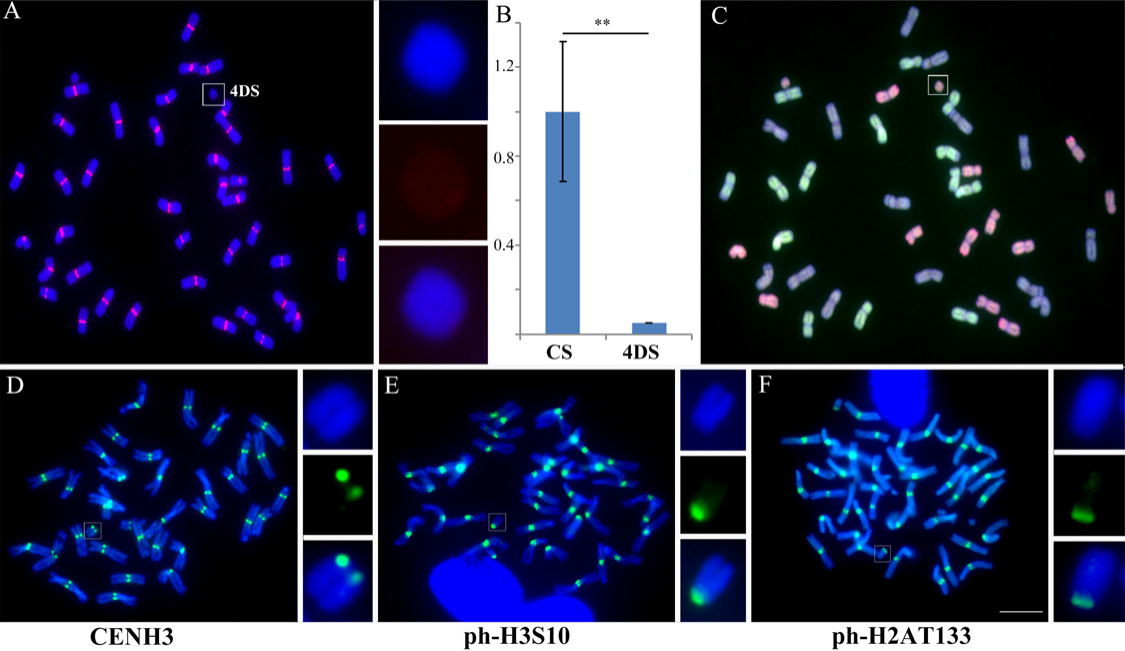
Centromeric sequence deletion in the ditelosomic line 4DS. (A). FISH on 4DS with CRW (red). DAPI staining is labeled in blue. (B). Intensity of CRW fluorescence in 4DS compared with the controls in Chinese Spring. The Y-axis represents the relative CRW fluorescence intensity. The error bars show the standard deviation (s.d.). The double asterisks denote significant differences at P<0.001 (two-tailed Student’s t-test). P = 3.59E-15. (C). Multi-color FISH on 4DS. The DNA for the wheat A genome is labeled in green, the DNA for the D genome is labeled in red, and the DNA for the B genome is used as a block. (D)-(F). Immunostaining results of 4DS with antibodies against CENH3 (D), H3 phosphorylation at Ser-10 (E), and H2A phosphorylation at Thr-133 (F) are shown in green. The insets show high-magnification images of chromosome 4DS. Bar = 10 μm.

Compared with normal centromeres in the *T*. *aestivum* Chinese Spring background, weaker fluorescence *in situ* hybridization (FISH) signals from the CRW probes were detected in the ditelosomic lines 5DL, 5DS and 1BS [55] (Fig 2A–2C, 2E–2G and 2I–2K and S1 Fig). Thus, significant reductions of centromeric sequences can frequently occur in allopolyploid wheat. However, CENH3 immunostaining revealed that functional centromeres were present in these three lines (Fig 2D, 2H and 2L). Additionally, in the ditelosomic line 4DS [55], we were unable to detect any CRW signals with FISH in the centromere or the chromosome arms, which stands in stark contrast to the normal chromosome 4D (Figs 3A–3C and S1). However, the epigenetic marks of active centromeres, including CENH3 and H2A phosphorylation at Thr-133 and H3 phosphorylation at Ser-10, were correctly loaded on the short arm of the 4D chromosome, suggesting that a *de novo* centromere had formed that lacked the canonical centromeric sequences (Fig 3D–3F).

**Fig 4:**
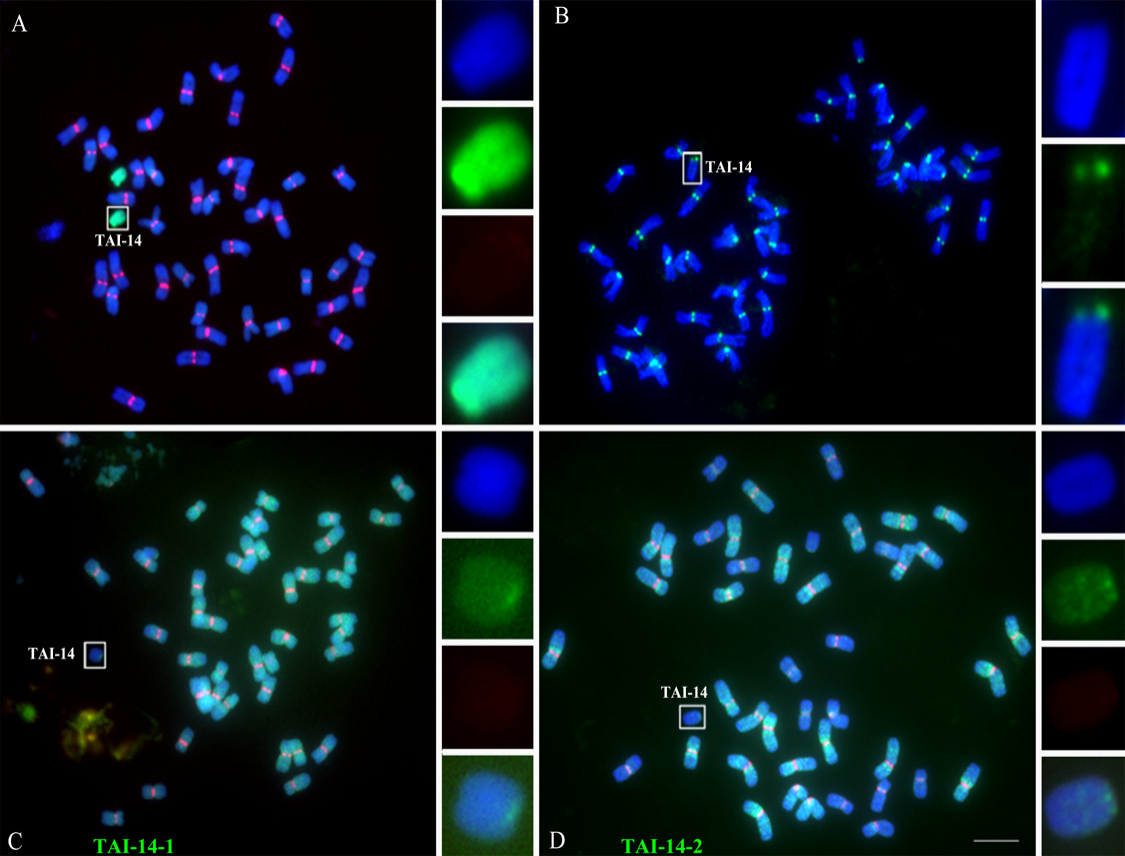
Deletion of centromeric sequences in the wheat-*Th*. *intermedium* addition line TAI-14. (A). Genomic *in situ* hybridization (GISH) and FISH of somatic metaphase chromosomes in TAI-14. The genomic DNA of *Th*. *intermedium* is labeled in green, the CRW sequences are labeled in red, and DAPI staining is labeled in blue. (B). Immunostaining of TAI-14 with antibodies against CENH3 (green). (C) and (D). FISH analysis of the novel centromeric sequences TAI-14-1 (C) and TAI-14-2 (D), which are labeled in green in TAI-14. The CRW sequences are labeled in red. The insets show high-magnification images of the alien chromosomes in TAI-14. Bar = 10 μm.

The wheat-*Th*. *intermedium* addition line TAI-14 was generated from hybrids between *T*. *aestivum* Xinshuguang 1 and amphidiploids zhong2 (2n = 56) [56]. The CRW sequences were heterogeneously distributed in the centromeric region of the 42 chromosomes of *Th*. *intermedium*, although some FISH-detected signals were very weak (S2A Fig). However, there were no detectable CRW signals on the *Th*. *intermedium*-derived chromosome in TAI-14 (Fig 4A). These chromosomes had functional centromeres, as revealed by the presence of CENH3 (Fig 4B).

**Fig 5:**
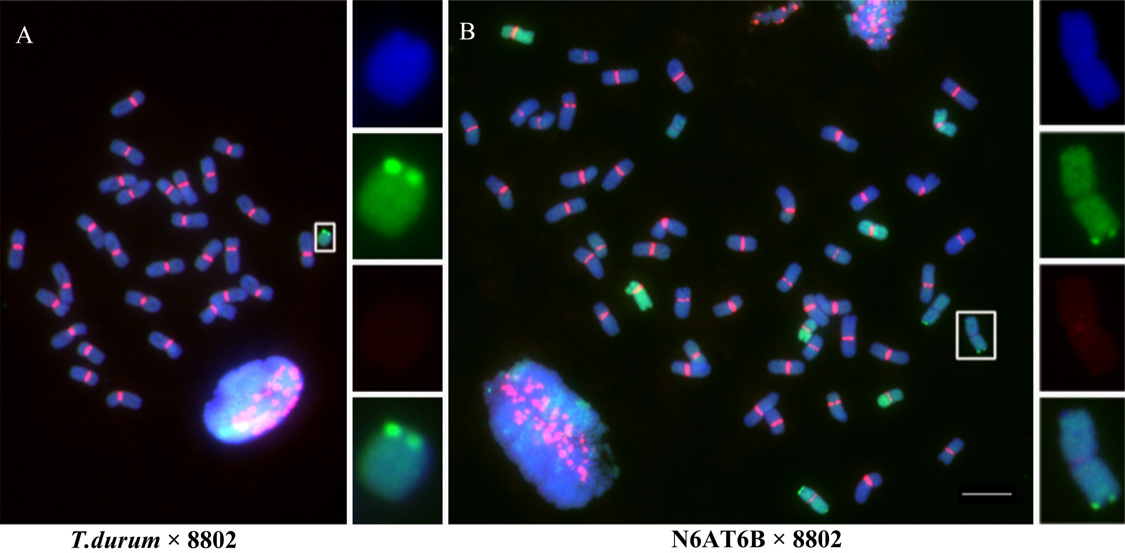
Deletion of centromeric sequences in the wheat-*Th*. *elongatum* addition lines. The genomic DNA of *Th*. *elongatum* is labeled in green, the CRW sequences are labeled in red, and DAPI staining is labeled in blue. (A) FISH analysis of a ditelosomic addition line from the hybrid of *T*. *durum* and 8802. There are no detectable FISH signals in the new telosomic chromosomes when CRW is used as a probe. (B) FISH analysis of an addition line from the hybrid of N6AT6B×8802 that shows weak CRW FISH signals. The insets show high-magnification images of the alien chromosomes with centromere changes from the addition lines. Bar = 10 μm.

Additionally, most copies of CRW in the two alien chromosomes from *Th*. *elongatum* were eliminated in the derivatives of the Chinese Spring nulli-tetrasomic lines N6AT6B (2n = 42) × wheat-*Th*. *elongatum* amphidiploid 8802 (2n = 42, AABBE1E2) (Fig 5B). The genome of 8802 consists of 28 chromosomes from *T*. *durum* Kekeruite (2n = 28) and 14 chromosomes from *Th*. *elongatum* AE31 (2n = 28) [57]. No obvious CRW FISH signals were detected on the chromosomes of *Th*. *elongatum* in the addition line derived from *T*. *durum* Kekeruite × 8802 (Fig 5A). However, the chromosomes that lack CRW sequences were stably transferred to the next generation, indicating that functional centromeres were formed on these chromosomes.

### Novel sequences are involved in *de novo* centromere formation on an alien chromosome

Without a reference genome sequence for *Th*. *intermedium*, it is difficult to characterize the sequences involved in the *de novo* formation of centromere in TAI-14. We designed a new strategy to isolate the neocentromere sequences based on CENH3-ChIP and dot-blot methods (see Methods). The CENH3-ChIP-enriched DNAs in the control Chinese Spring (abbreviated as CS) and TAI14 were further analyzed by dot-blotting. The signals from the dot-blots that were significantly different between CS and TAI14 (e.g., signals present in TAI14 and not in CS) were treated as potential elements involved in *de novo* centromere formation. Two sequences, TAI-14-1 and TAI-14-2, were identified as new centromeric sequences for the new centromere in TAI-14 (Fig 4C and 4D), and both sequences showed homology to known retrotransposons by alignment to a BAC genome sequence in wheat (S3 Fig). TAI-14-1 was widely dispersed on nearly all of the chromosomes, whereas TAI-14-2 was mainly detected in the pericentromeric regions of the wheat chromosomes (Fig 4C and 4D). We observed that TAI-14-2 was located at both the centromeric and pericentromeric regions on different chromosomes in *Th*. *intermedium* (S1B Fig). Interestingly, some chromosomes of *Th*. *intermedium* that showed less CRW distribution were accompanied by more TAI-14-2 sequences occupancy in the centromeric region (S2D and S2E Fig). This result suggests that CRW and TAI-14-2 may be complementary centromeric sequences in some chromosomes of *Th*. *intermedium*.

### A 994-kb genomic sequence of the 4DS chromosome is involved in *de novo* centromere formation in 4DS

The total loss of CRW sequences and the presence of CENH3 in the 4DS ditelosomic line suggest that a new centromere formed on the 4DS chromosome (Fig 3). Because sequenced genomes published for *Ae*. *tauschii* (wheat D genome donor) and CS [58,59], we performed a ChIP-seq analysis with CENH3 antibodies and identified potential sequences in the new centromere on the 4DS chromosome. Because the genomes were not completely assembled, we chose a mapping strategy that equally mapped all reads to multiple loci. The raw reads of the 4DS and CS (as control) samples were mapped to the wheat D and CS genomes, respectively, using BWA software (S1 Table) [60].

**Fig 6:**
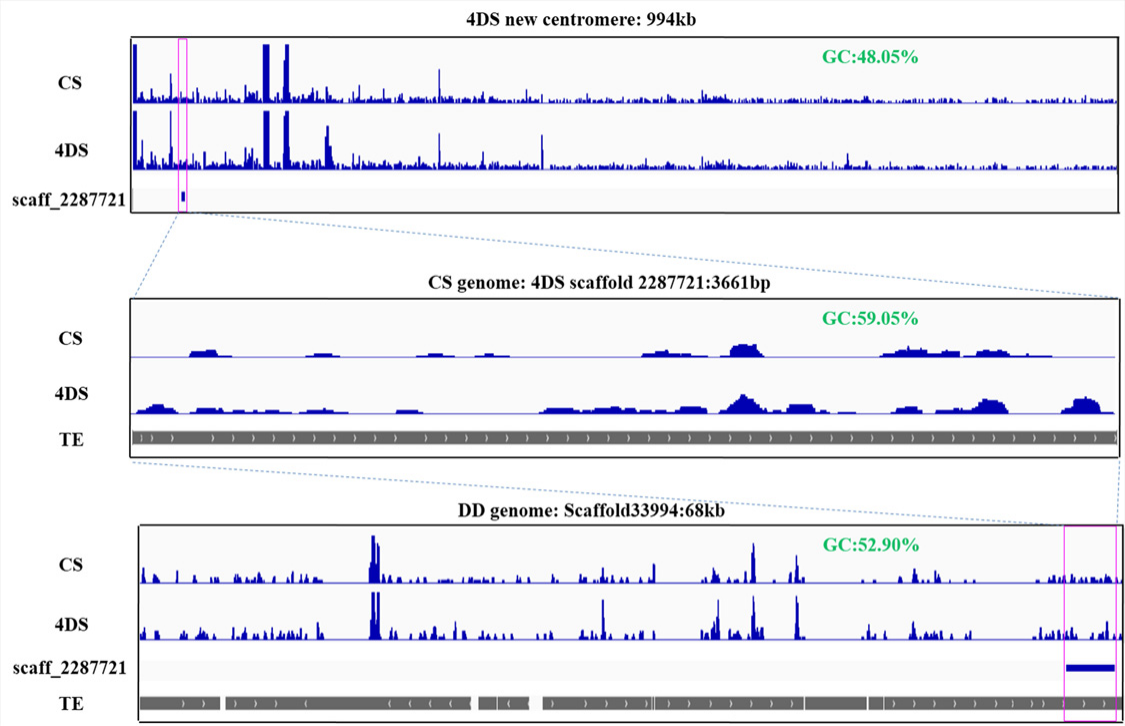
ChIP-seq mapping results of the ditelosomic line 4DS with the CENH3 antibody. The top box shows the ChIP-seq results of the control Chinese Spring (CS) and 4DS by mapping to the CS genome. A 994-kb region was detected as a difference between CS and 4DS and is involved in 4DS neocentromere formation. 4DS Scaffold 2287721 (3,661 bp, indicated by the magenta box) was selected as a probe for FISH confirmation. The scaffold was enriched with transposable elements, as shown in the middle box. In the wheat D genome, Scaffold 33994 (~68 kb, anchored to chromosome 4D) has high homology with Scaffold 2287721 and shows a mapping difference between CS and 4DS. The 994-kb region, Scaffold 2287721 and Scaffold 33994 had high GC contents.

**Fig 7:**
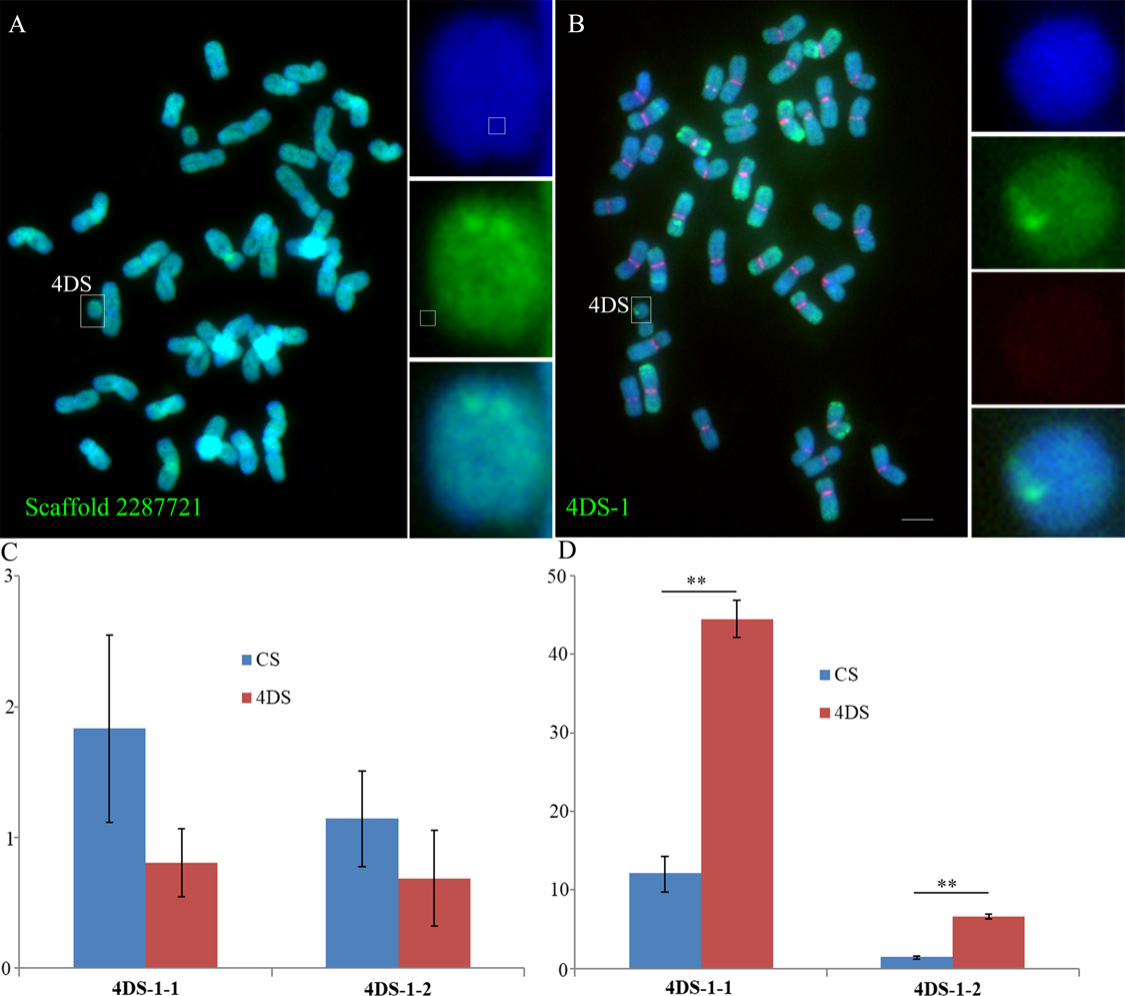
FISH of a novel centromeric sequence in 4DS identified by ChIP-seq mapping and dot-blot hybridization. (A). Scaffold 2287721 (labeled in green), which was obtained from ChIP-seq, contained sequences of the *de novo* centromere in 4DS. (B). 4DS-1 (labeled in green), which was obtained from the dot-blot, was detected as a novel centromere sequence in 4DS. The CRW sequences are labeled in red. The insets show high-magnification images of 4DS. Bar = 10 μm. (C). RT-qPCR analysis of 4DS-1-1 and 4DS-1-2. (D). The RNA-ChIP-qPCR results for 4DS-1-1 and 4DS-1-2 suggest that transcription levels of both sequences at the 4DS centromeres were increased.

Using the sequence of the CS genome as a reference, we identified 107 scaffolds on the short arm of the 4D chromosome, with different CENH3 enrichments between 4DS and CS. The sizes of the 107 scaffolds ranged from 1,594 to 32,269 bp, and these scaffolds were combined into a 994-kb region containing only 11 genes that code for ribosomal and photosystem proteins (Fig 6, S2 Table). Furthermore, we selected one of the assembled scaffolds, IWGSC_CSS_4DS_scaff_2287721 (3665 bp), as a FISH probe and confirmed its localization in the 4DS *de novo* centromere (Fig 7A). We mapped this scaffold to the genome of *Ae*. *tauschii* using BLASTN [61] and identified a 68-kb fragment (Scaffold 33994) that contained most of the sequences of the scaffold that showed mapping differences between the 4DS and CS (Fig 6). Both of the sequences, the homologous 3,665-bp sequence from the CS genome and the 68-kb sequence from the D genome, contained many transposable elements and similar GC levels (48.05% and 52.90%, respectively, Fig 6). Due to the incomplete genome sequence, we tentatively suggest that the partial sequences of the 994-kb region in the wheat CS genome and the 68-kb region in wheat D genome may underlie *de novo* centromere formation in 4DS.

In addition, the same strategy that was used to isolate the neocentromere sequences on TAI-14 was employed with 4DS to better understand the sequences in the 994-kb region. A 769-bp fragment (named 4DS-1) near the original centromere was identified as a candidate sequence in the 4DS *de novo* centromere (Figs 7B and S4A). 4DS-1 was present at multiple locations on the chromosomes from the A and D genomes. FISH detection showed that it was localized to sites near the normal centromere on the 2A, 7A, 7D and 2D chromosomes (S4B and S4C Fig). However, FISH signals of 4DS-1 were not observed in the chromosomes from the B genome (Figs 7B and S4). 4DS-1 was mapped to two scaffolds (Scaffold 10770 and Scaffold 28550) from the 4DS chromosome and showed mapping differences between 4DS and CS, which confirmed that 4DS-1 was a part of the *de novo* centromeric sequence in the 4DS ditelosomic line (S5 Fig).

### Transcription and histone modification accompanied *de novo* centromere formation in 4DS

A previous study showed that the transcripts of centromeric sequences can function as essential components of centromere structure and activity [37]. Histone modifications, such as methylation and phosphorylation, are important regulators of centromere stability and activity [35,38]. For sequence 4DS-1 in the 4DS *de novo* centromere, we tested whether there were changes in transcription and the transcripts that interacted with CENH3 via RT-qPCR and RNA-CENH3-ChIP. We selected two fragments (~300 bp) of the 4DS-1 sequence, termed 4DS-1-1 and 4DS-1-2. Compared with CS, the transcripts of 4DS-1-1 and 4DS-1-2 were slightly but not significantly decreased in 4DS (Fig 7C). However, the amounts of the 4DS-1-1 and 4DS-1-2 transcripts that were associated with CENH3 were remarkably increased in 4DS compared with CS (Fig 7D). These results suggest that increased transcription of the corresponding sequences may accompany *de novo* centromere formation in 4DS. We also checked the possible changes in six histone modifications between the normal and *de novo* centromeres via immunoassay. No significant signals for the euchromatin marks H2AZ and H3K4me3 were detected in either centromere (S6A and S6B Fig), and enrichment of the euchromatin-related histone mark H3K4me2 was discernible for both centromeres (S6C Fig). Compared with non-centromeric chromosome ends, both centromere types revealed a reduction in the heterochromatin marks H3K27me2 and H3K27me3 (S6D and S6E Fig), whereas there were no obvious differences in H3K9me2 in the two centromere types (S6F Fig). In general, there were no significant differences in the accumulation of most euchromatic or heterochromatic histone markers between the normal and *de novo* centromeres.

### Multi-locus centromere formation in wheat wide hybrids

**Fig 8:**
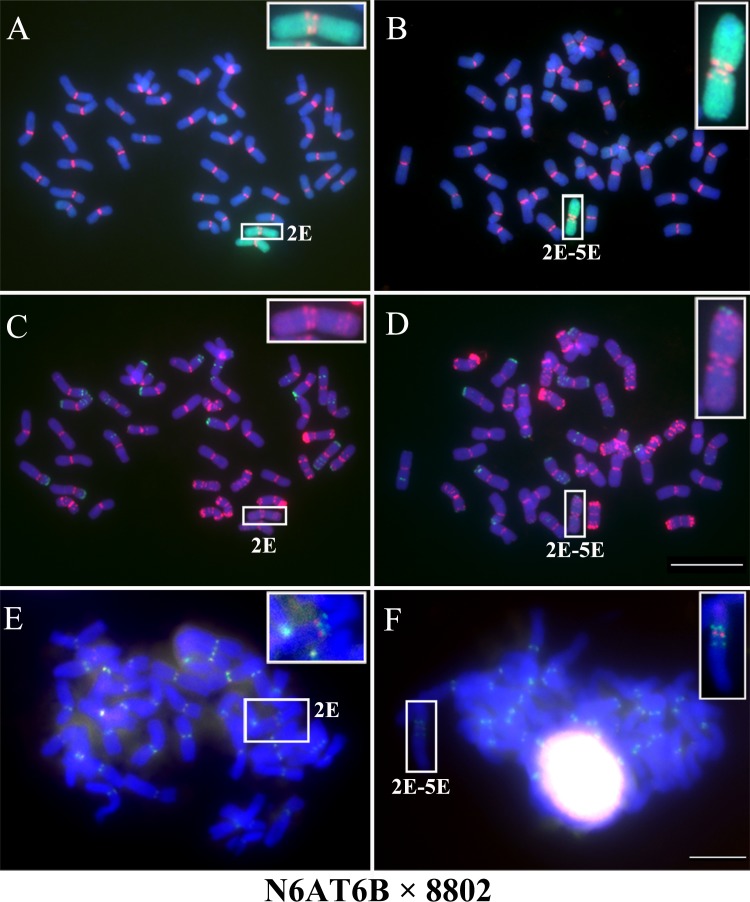
Two- and three-locus centromeres in the F5 generation of hybrids between nulli-tetrasomic N6AT6B× 8802. (A) and (B). FISH and GISH of chromosomes with two- and three-locus centromeres, respectively. The genomic DNAs of *Th*. *elongatum* are labeled in green, the CRW sequences are labeled in red. DAPI staining is labeled in blue. (C) and (D). Karyotype analysis of chromosomes with two- and three-locus centromeres using probes pAsI (green) and pSc119.2 (red). (E) and (F). Immunostaining-FISH analysis of chromosomes with two- and three-locus centromeres. The CRW sequences are labeled in green, and CENH3 is labeled in red. The insets show high-magnification images of the chromosomes with two- and three-locus centromeres. Bar = 10 μm.

We crossed the hexaploid amphidiploid 8802 with the *T*. *aestivum* Chinese Spring nulli-tetrasomic lines to establish new substitution lines for the chromosomes from the E genome. All chromosomes from 8802 have only one centromere, as determined by the FISH signals of CRW (S7A Fig). Chromosomes with two regions containing CRW sequences were identified in the F1 hybrids of the nulli-tetrasomic (3, 5 and 6 homologous groups) lines × 8802 (S7C–S7F Fig). In the F5 generation of the hybrids between nulli-tetrasomic line N6AT6B (2n = 42) × 8802, chromosomes containing two regions with centromeric sequences were inherited from the F1 generation, but only one region was functional (Fig 8A and 8E). In addition to the two-locus centromere, a three-locus centromere was discovered on an alien chromosome in the F5 generation (Fig 8B), and the middle centromere region was shown to act as the functional centromere (Fig 8F). Furthermore, two different three-locus centromeres were observed in the F6 generation (S8C and S8D Fig).

The repetitive sequences pAs1 and pSc119.2 were used to karyotype the chromosomes with two- or three-locus centromeres. The chromosomes with two centromeric regions originated from the 2E chromosome of 8802, but the three centromeric regions were produced by the combination of sequences from two different arms of chromosomes 2E and 5E, rather than direct inheritance from any chromosome (Figs 8B, 8D and S7B). Furthermore, two different three-locus centromeres were observed in the progeny. One progeny contained an isochromosome 2ES, and the other progeny contained a chromosome produced from the wheat 6BL chromosome and the *Th*. *elongatum* 2E chromosome (S8A and S8B Fig). However, neither was stably transferred to the next generation.

Multi-centric chromosomes are frequently formed in the hybrids of wheat and related species, such as *Th*. *elongatum*, *Th*. *poticum*, *Th*. *intermedium*, *Agropyron cristatum*, *Hordeum vulgare* and *S*. *cereale* (S9 Fig). However, unlike the two-locus centromere in the hybrids of N6AT6B × 8802, both centromeres in these dicentric chromosomes were active, which caused chromosomal loss in the next generation.

### Expansion of centromeric retroelements in wheat-rye addition lines

Heterochromatin alterations and chromosomal rearrangements associated with centromere changes have been reported in derivatives of wheat-rye hybrids [62]. The chromosomes containing altered centromeres were lost in the next generation. Here, we discovered another wheat-rye hybridization-promoted centromeric retrotransposon expansion in different rye addition lines. These changes can be stably transmitted to offspring.

**Fig 9:**
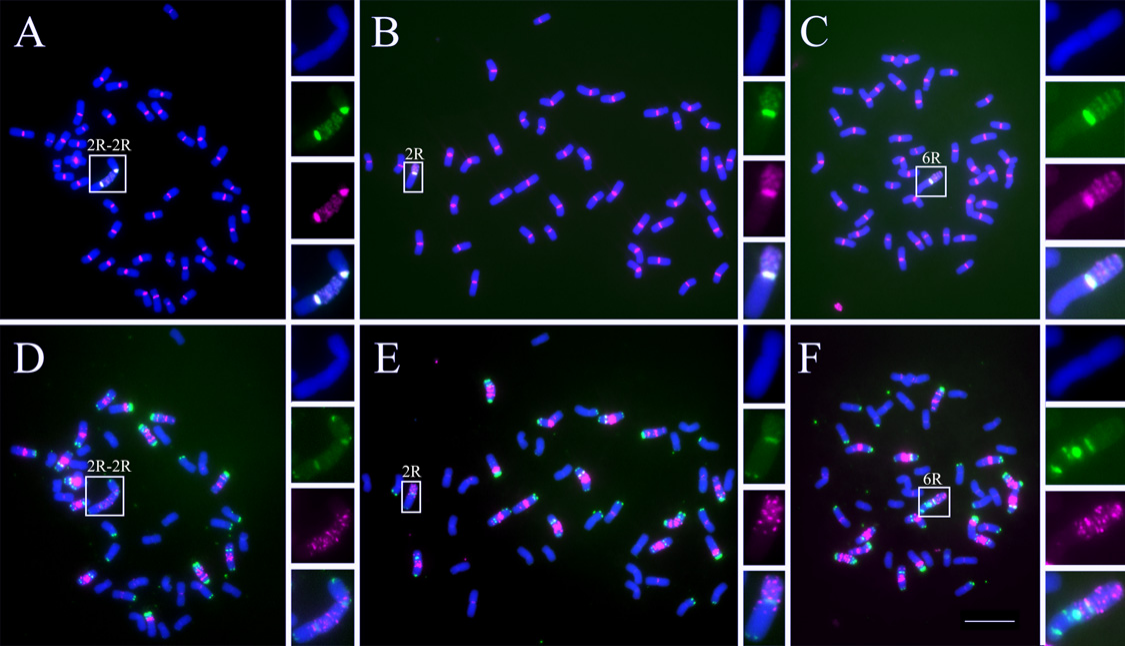
Centromere expansion in the wheat-rye addition lines. (A)-(C). FISH indicates centromere expansion in the 2R-2R, novel 2R and 6R addition lines using the pAWRC.1 (green) and CRW (red) probes. (D)-(F). Karyotype analysis of centromere expansion in the 2R-2R (D), novel 2R (E) and 6R (F) addition lines using the AAC (red) and pSc119.2 (green) probes. The insets show high-magnification images of the chromosomes with expanded centromeres. Bar = 10 μm.

The rye addition lines were generally obtained from successive backcrossing between wheat and triticale. A novel octoploid triticale (2n = 56) was generated by hybridization between *T*. *aestivum* Mianyang 11 (2n = 42) and *S*. *cereale* Kustro (2n = 14). In the wheat and octoploid triticale hybrids, a novel chromosome emerged after the joining of the two 2R chromosomes (2R-2R). Compared with the normal centromeres of chromosomes 2R and 2RL, the centromere in the 2R-2R chromosome was drastically expanded and was much larger than the 2RL arm (Figs 9A, 9D and S10). Further analysis showed that this large centromere consisted of two normal centromeres from chromosome 2R and a centromere-like region between them with dispersed pAWRC.1 (rye-specific centromeric retrotransposon) sequences [63] (Fig 9A). Dispersed centromeric retrotransposons may function as a part of an active centromere, as 2R-2R was broken into smaller fragments after self-pollination (Fig 9B and 9E). In the progeny, we detected a new 2R chromosome (smaller than the canonical 2R) that retained a region with dispersed pAWRC.1 sequences and was approximately half the size of 2R-2R (Figs 9B, 9E and S10B). A novel chromosome 6R contained pAWRC.1 sequences in a region near the functional centromere in the 6R addition line (Figs 9C, 9F and S10B). However, these regions did not have centromere activity in these progeny (S11 Fig).

## Discussion

### *De novo* centromere formation adjacent to native centromeres depends on unique epigenetic modifications in wheat and its wide hybrid

Functional centromeres without classic centromeric sequences have been reported in humans, fungi, and plants [29–32,64,65]. Here, we found that most neocentromeres in wheat and its addition lines consist of genomic sequences that have loose resemblance to the sequences of normal centromeres. The sequences involved in the *de novo* centromere formation in the ditelosomic lines 4DS and TAI-14 were located at the chromosome arms adjacent to the native centromere. The 4DS-1 sequence was located very near the centromeres of chromosomes 4D, and sequence TAI14-2 was detected in the pericentromeric region before the new centromere had formed (Figs 7B and S4). The preferential localizations of the *de novo* centromeres in wheat and its addition lines were similar to some of the newly formed centromeres in chicken, *Candida albicans* and a wheat-barley addition line [66,67]. However, in contrast to wheat, human neocentromeres can originate at multiple positions on different chromosomes through the binding of essential kinetochore proteins [30]. In addition, neocentromeres also have been observed at multiple locations on chromosome arms in other plants [29,32,65]. This difference suggests that the *de novo* centromere formation was not dependent on location and occurred under appropriate epigenetic conditions, as indicated by previous studies [28,31,32].

We observed that CENH3, H2AT133ph and H3S10ph were deposited in all of the newly formed centromeres in wheat and its wide hybrids (Figs 2D, 2H, 2L, 3D–3F and 4B). With the exception of CENH3 or CENP-A, other histone modifications in the centromeric region are also critical elements for centormere stability and activity. CENP-A nucleosomes are interspersed with the H3K4me2 nucleosomes within the centromeric chromatin of humans and flies [68,69]. Enrichment of H3K9me2 and H3K9me3, but not H3K4me2, has been observed in maize centromeres [70,71]. Furthermore, H3K4me2, H4ac and H3K27me1 were enriched in the centromeric chromatin of rice [72]. We observed that H3K4me2 was slightly enriched in the normal and newly formed centromeres of wheat, similar to previous reports in humans, flies and rice. However, weak signals for H3K27me and H3K9me2 were observed in these two centromeres, which were different from the centromeres of maize and rice (S6 Fig). Based on our results, the histone modifications in the new centromeres are similar to the normal centromeres, highlighting that possibility that unique histone modifications in wheat centromeric regions may be required for *de novo* centromere formation and stability.

Noncoding RNAs from the centromeric sequences directly interact with the kinetochore and recruit CENPC to centromeres [73]. In maize, transcripts of centromeric retrotransposons and repeat sequences have been associated with the CENH3 protein, as detected by ChIP with anti-CENH3 antibodies [74]. Our results showed that the expression of neocentromeric sequence 4DS-1 was greatly elevated during the process of *de novo* centromere formation (Fig 7C and 7D). The transcripts of centromeric sequences may serve as structural and regulatory components of the centromere. In fact, its transcription process, rather than the transcripts themselves, may have facilitated CENP-A deposition and nucleosome assembly at the centromere [35,37]. *De novo* centromere formation in 4DS increased the expression of the 4DS-1 sequence, which may provide the RNAs that regulate CENH3 deposition and recruit epigenetic elements [35,37].

### Highly variable of new centromeric sequences between different chromosomes and species

For cereal centromeres, two common sequences, described as *cereal centromeric sequence* (CCS1) and Sau3A*9*, were first reported in *Brachypodium* and *Sorghum bicolor* [75,76]. In most cereals, these centromeric sequences represent parts of the *Ty3*/*gypsy* retroelement, e.g., ‘*cereba’* of barley, implying that cereals maintain conserved retroelements in their centromeric regions [77]. However, the tandem repeats in the centromere are species-specific [77]. We observed that the pericentromeric sequence TAI-14-2 of wheat was located in the centromeric region of several chromosomes of the wild relative *Th*. *intermedium* (S2 Fig). The centromeric sequences may undergo insertion and amplification during species differentiation [20,25,51], providing insight into the mechanism of differential centromeric sequences change in wheat and its wild relatives.

Centromeres on different chromosomes of one species may evolve rapidly and are independent of each other [20]. The novel centromeric sequence 4DS-1 displayed a specific localization pattern in the different chromosomes of the wheat subgenomes, and its chromosomal location was very near the centromeres of chromosomes 7A, 7D, 2A and 2D (Figs 7B and S4). However, the sequence was not readily detectable on chromosomes 2B and 7B, and it was not found in other chromosomes, such as 4A and 4B. Similar to the situations in rice, maize, and potato, the centromeres on different chromosomes experienced differentiation by sequence loss and insertion [20,25,51].

Centromeric protein complexes, which are necessary for proper chromosome segregation, can be formed by the speciation factors HMR and LHR to mediate hybrid sterility and incompatibility in *Drosophila* [78]. During hybridization, the intragenomic conflicts of different centromeres may cause incompatibilities in hybrids [79,80]. These results strongly suggest that centromere divergence has an important impact on the generation of biodiversity [78]. To adapt to highly variable centromere DNA sequences, the centromere proteins undergo rapid evolution to maintain a functional centromere [81]. We observed that the new centromeric DNA sequences are highly variable between different genomes and chromosomes, which may allow the centromeric proteins to mediate intragenomic incompatibility and genomic specificity in the nascent hybrids. Although detailed reports are not available, it is likely that centromere sequence diversity has an important impact on speciation. An understanding of the diversity of centromeric sequences and its link to speciation and genomic stability deserve further analysis.

### Most variations in the centromeric sequences occur during wide hybrid formation

Multiple centromeres, holocentromeres and neocentromeres formation implies that centromere positions may be alterable rather than permanently fixed [11,31,32,82–84]. Wide hybridization can trigger chromosomal rearrangements and genome reorganization, accompanied by centromere alterations [62,85]. Unstable di-centromeres and multiple centromeres have been associated with the formation of inter- and intra-chromosomal translocations in wheat-rye hybrids [62]. Our results demonstrated that multi-locus centromere formation, centromere expansion, and canonical centromeric sequence elimination may yield novel chromosomes in wheat and its wide hybrids (see summary Fig 1).

Chromosomes with two regions containing centromeric sequences were observed in the F1 hybrids of three null-tetra lines and 8802 (S7 Fig). During meiosis I of the F1 generation, several univalents of the E genome in amphidiploid 8802 may experience centromere breakage. Centromere misdivision, which depends on the orientation of the univalent, may occur across the either centromere or the pericentric chromatin, but chromosome fragments containing centromeric and pericentric regions may survive [86]. This observation suggests the possibility that chromosomal fragments containing centromeric and pericentromeric regions were rejoined to a novel chromosome and induced the formation of two- and three-locus centromeric regions. Centromere inactivation allows dicentric chromosomes with only one functional centromere to be stably transmitted to the next generation (Fig 8E), similar to stable dicentric A-A and A-B chromosomes in maize [18,33,34]. However, the chromosome containing a three-locus centromere still suffered from centromere breakage, which led to its structural alterations in the progeny. As a consequence of a translocation in the nulli-tetrasomic line N6AT6B, a chromosome with a three-locus centromere included the 2E chromosome from 8802 and the 6B chromosome from N6AT6B (S8D Fig). Similar to rye and maize [18,86,87], intrachromosomal recombination and centromere breakage likely promoted the formation of multi-locus centromeres and novel chromosomes in wheat and its wide hybrids.

Retrotransposons can be activated during wide hybridization [88]. Interspecific hybrids triggered the amplification of centromeric satellite repeats and retrotransposons [85]. In a wheat-rye hybrid, we observed two chromosomes that had likely lost their telomeres and fused into one chromosome, as previously suggested [89]. The wide hybridization affected the stability of the centromeric retrotransposons and the activation of rye-specific retrotransposons in the 2R-2R and 6R addition lines, causing centromeric sequence expansion (Fig 9A and 9C). These unstable centromeres may subsequently lead to chromosome breakage and different progeny with expanded centromeres, suggesting that centromere variants may trigger the formation of novel chromosomes. Total centromere size has been postulated to be positively correlated with genome size rather than chromosome size [90]. Centromere domains of several maize chromosomes (average size 1.8 Mb) expanded to 3.6 Mb in the background of the oat genome [48]. However, the expanded centromere on chromosome 2R-2R may instead be a general response to genomic stress following the wheat-rye hybridization, rather than an adaptation to the wheat centromere size.

In summary, we observed that the elimination, rearrangement and expansion of centromeric sequences affect chromosome morphology and maintenance in wheat wide hybrids. *De novo* centromere formation promotes the accurate segregation of chromosomes that experienced centromere sequence elimination. The new centromeres have low sequence homology but high epigenetic similarity to normal centromeres. The highly variable centromeric sequences between genomes and chromosomes may facilitate genomic specificity and differentiation in hybrids. The centromeric sequences involved in *de novo* centromere formation are mainly retrotransposon-like sequences, and their RNAs are transcribed at high levels. Multiple centromeres and centromere sequence expansion strongly influence centromere activity and cause chromosome breakage and rearrangements. More importantly, centromere variations in the ditelosomic lines 4DS, 1BS, 5DL, 5DS and TAI-14 may specifically affect the size and DNA organization of normal chromosomes. Thus, these may be useful for future studies of chromosome sorting and sequencing.

## Methods

### Plant materials

The hexaploid amphiploid 8802 (AABBEE) originated from hybrids between *T*. *durum* and *Th*. *elongatum* [57]. The addition lines of wheat (Chinese Spring)-*Th*. *poticum*, *Th*. *intermedium* and *Agropyron cristatum* were produced by our laboratory. The addition lines of wheat (Mianyang 11) and *S*. *cereale* (Kustro) were produced by Dr. Shulan Fu, Sichuan Agriculture University, Chengdu, China. The nulli-tetrasomic lines and the ditelosomic lines 4DS, 1BS, 5DL and 5DS were kindly provided by Dr. Perry Gustafson, University of Missouri, Columbia, MO, USA.

### Cytological preparation, probe labeling and images processing

The root tips were prepared for the FISH experiments and probes as previously described [91]. The CRW and other genomic DNAs were labeled with Alexa Fluor-488-dUTP (green) or Alexa Fluor-594-5-dUTP (red) as needed. The root tip samples of the different wheat lines were treated with the same conditions, and with an equal amount of probe. The FISH images were acquired using an epifluorescence Olympus BX61 microscope (Olympus China Inc, Beijing, China) with the same exposure time and were processed with Adobe Photoshop CS 3.0. For analysis, the fluorescence was quantified using ImageJ [92]. At least 20 cells from three different plants were counted in each of the different wheat lines. Significant differences were calculated using Microsoft Excel and Student’s t-test (two-tailed).

### Immunolocalization in somatic cells

The root tips were fixed with 4% formaldehyde in 1×PBS for 1 h, and the metaphase chromosomes were prepared as previously described [17]. The wheat-specific CENH3 antibodies were produced in our laboratory. The phH2AThr-133 and phH3Ser-10 antibodies were described previously [16,17].

### ChIP-seq, RNA-ChIP-qPCR and RT-qPCR

Chromatin immunoprecipitation (ChIP) was performed according to a previously described method [93]. Approximately 20 g of fresh leaf tissue from Chinese Spring and 4DS was prepared for CENH3-ChIP (CENH3 antibody used as described above). ChIP-seq was conducted according to a previously described method [32]. Using an Illumina HiSeq2000 platform, the enriched DNA samples were sequenced to generate paired-end 100-bp sequence reads. RNA-ChIP was performed using a method that was similar to ChIP [93]. RNase activity was inhibited using Recombinant RNase inhibitor (RRI) in the RNA-ChIP process. The RNA was extracted from the sample using TRIzol reagent. The RNA was reverse-transcribed into cDNA using M-MLV reverse transcriptase (Promega) and random primers (New England Biolabs). The qPCR protocol was performed as described [94].

### ChIP-seq data analysis

Nearly 14,000 Mbp of raw ChIP-seq paired-end 100-bp reads were mapped to the wheat CS and wheat D genomes using BWA software [60]. All mappable reads were randomly assigned a locus from the possible options, and the duplicated reads were removed. The reads per million (RPM) values of every scaffold in the genome were calculated to show the normalized enrichment. We selected the scaffolds that had enriched reads in both 4DS and the control, with the RPM ratios between two samples ≥20, or scaffolds that only had enriched reads in 4DS, with read counts ≥20. IGV Tools was used to visualize the normalized read distribution of each scaffold [95]. The anti-CENH3 ChIP-seq data were deposited in the Gene Expression Omnibus (GEO) database under number GSE63752.

### Dot-blot hybridization of the ChIP-enriched DNAs

The ChIPed DNAs from CS and 4DS were amplified by Dop-PCR (primer sequence was 5’-CCGACTCGAGNNNNNNATGTG G-3’) [96]. The DOP-PCR product was used as the DNA target, and the ChIPed DNAs were used as probes. The dot-blot protocol was performed as described [97]. The differences in the sequences between CS and 4DS that were determined by the dot-blot hybridization were selected as candidate centromeric sequences for FISH.

### Wheat genetic nomenclature

#### Addition line

An individual with addition chromosomes from other species.

#### Ditelosomic line

An individual with a pair of chromosome arms.

#### Nulli-tetrasomic line

An individual lacking one pair of chromosomes and containing a doubled pair of other chromosomes.

## Supporting Information

S1 Fig Centromeric sequences in the different chromosomes of Chinese Spring. (A) FISH of the CS chromosomes with CRW (red). DAPI staining is labeled in blue. (B) Karyotype analysis of CS using the pAsI (green) and pSc119.2 (red) probes. DAPI staining is labeled in blue. Bar = 10 μm. The 1B, 4D and 5D chromosomes are indicated by green, white, and red arrows, respectively.
(TIF)

S2 Fig Novel centromeric sequences, TAI-14-2, on chromosomes in *Th*. *intermedium*. (A)-(C) FISH of TAI-14-2 on chromosomes in *Th*. *intermedium*. The CRW sequences are labeled in red, TAI-14-2 is labeled in green, and DAPI staining is labeled in blue. Bar = 10 μm. (D)-(E) Line profile plots of the distributions of the intensity of the CRW and TAI-14-2 signals along the white line of selected chromosomes from (C).
(TIF)

S3 Fig Alignment of TAI-14-1 and TAI-14-2 DNAs to the BACs of wheat chromosome 3B. (TIF)

S4 Fig Cytological analysis of 4DS-1 in Chinese Spring and the ditelosomic line 4DS. (A) FISH of 4DS-1 on chromosomes in Chinese Spring. The CRW sequences are labeled in red, 4DS-1 is labeled in green, and DAPI staining is labeled in blue. The arrows indicate the 4D chromosomes. (B) Karyotype analysis of 4DS-1 in 4DS using the pAsI (green) and pSc119.2 (red) probes. DAPI staining is labeled in blue. The 2A, 7A, 2D, 4D and 7D chromosomes are indicated in the figure. (C) Multi-color GISH of line 4DS. The DNA for the wheat A genome is labeled in green, the DNA for the D genome is labeled in red, and the DNA for the B genome is used as a block. Bar = 10 μm.
(TIF)

S5 Fig Mapping results of the novel centromeric sequence 4DS-1 to the wheat D genome. The novel centromeric sequence 4DS-1 shows mapping differences between CS and 4DS in Scaffolds10770 and 28550, as indicated by the magenta boxes.
(TIF)

S6 Fig Histone modifications on the 4DS *de novo* centromere and a randomly selected centromere (control). The immunostaining with antibodies against (A) H2AZ; (B) H3K4me2; (C) H3K4me3; (D) H3K27me2; (E) H3K27me3; and (F) H3K9me2 –labeled in green. CENH3 is labeled in red. Bar = 10 μm.
(TIF)

S7 Fig FISH on the 8802 and F1 hybrids between nulli-tetrasomic lines and 8802. (A) Chromosomes of 8802. The genomic DNA of *Th*. *elongatum* is labeled in green, the CRW sequences are labeled in red, and DAPI staining is labeled in blue. (B) Karyotype analysis of 8802. pAsI is labeled in green, and pSc119.2 is labeled in red. The arrows indicate the 2E chromosome. (C)-(F) FISH analysis of the F1 hybrids of N3AT3B×8802 (C), N5BT5A×8802 (D), N5DT5B×8802 (E) and N6AT6B×8802 (F). The insets show high-magnification images of the chromosomes with two centromeric regions. The arrows indicate the chromosomes with changes in their centromeres. Bar = 10 μm.
(TIF)

S8 Fig Chromosomes with three-locus centromeres in the F5 generation of hybrids between nulli-tetrasomic N6AT6B × 8802. (A) and (B). Karyotype analysis of two different progeny of the three-locus centromeres using the pAsI (green) and pSc119.2 (red) probes. (C) and (D). FISH and GISH on two different progeny with chromosomes bearing a three-locus centromere. The genomic DNA of *Th*. *elongatum* is labeled in green, the CRW sequences are labeled in red, and DAPI staining is labeled in blue. The insets show high-magnification images of the chromosomes with three-locus centromeres. Bar = 10 μm.
(TIF)

S9 Fig Dicentric and multi-centric chromosomes in hybrids of wheat and wild relatives. (A) FISH and GISH of the dicentric and multi-centric chromosomes in hybrids of wheat and *Th*. *elongatum*. The DNA of *Th*. *elongatum* is labeled in red, the CRW sequences are labeled in green, and DAPI staining is labeled in blue. In (B) and (C), the CRW sequences are labeled in red. (B) FISH of the dicentric and multi-centric chromosomes in hybrids of wheat and *Th*. *poticum*. (C) FISH of the dicentric chromosomes in hybrids of wheat and *Th*. *intermedium*, *Agropyron cristatum*, *Hordeum vulgare* and *S*. *cereale*. Bar = 10 μm.
(TIF)

S10 Fig Cytological analysis of *S*. *cereale* Kustro and its 2R and 2RL addition lines. (A) FISH of *S*. *cereale* Kustro with the CRW (red) and pAWRC.1 (green) probes. (B) Karyotype analysis of *S*. *cereale* Kustro using the AAC (red) and pSc119.2 (green) probes. (C) and (D) FISH analysis of the 2R and 2RL addition lines. The genomic DNA of rye is labeled in green, the CRW sequences are labeled in red, and DAPI staining is labeled in blue. Bar = 10 μm.
(TIF)

S11 Fig Immunodetection of CENH3 and GISH with rye DNA in the novel wheat-rye 2R and 6R addition lines. (A) and (B). The novel 2R and 6R addition lines, respectively. The genomic DNA of rye is labeled in green, CENH3 is labeled in red, and DAPI staining is labeled in blue. The insets show high-magnification images of the chromosomes with expanded centromeres. Bar = 10 μm.
(TIF)

S1 Table Statistics of the ChIP-Seq mapping results. (DOCX)

S2 Table Detailed scaffolds and genes located in 994-kb region of the new centromere in 4DS. (XLS)
