# Supplementary figures and images for: De Novo Centromere Formation and Centromeric Sequence Expansion in Wheat and its Wide Hybrids
Source: PLoS Genet. 2016 Apr 25;12(4):e1005997. doi: 10.1371/journal.pgen.1005997 (PMC4844185; doi:10.1371/journal.pgen.1005997)

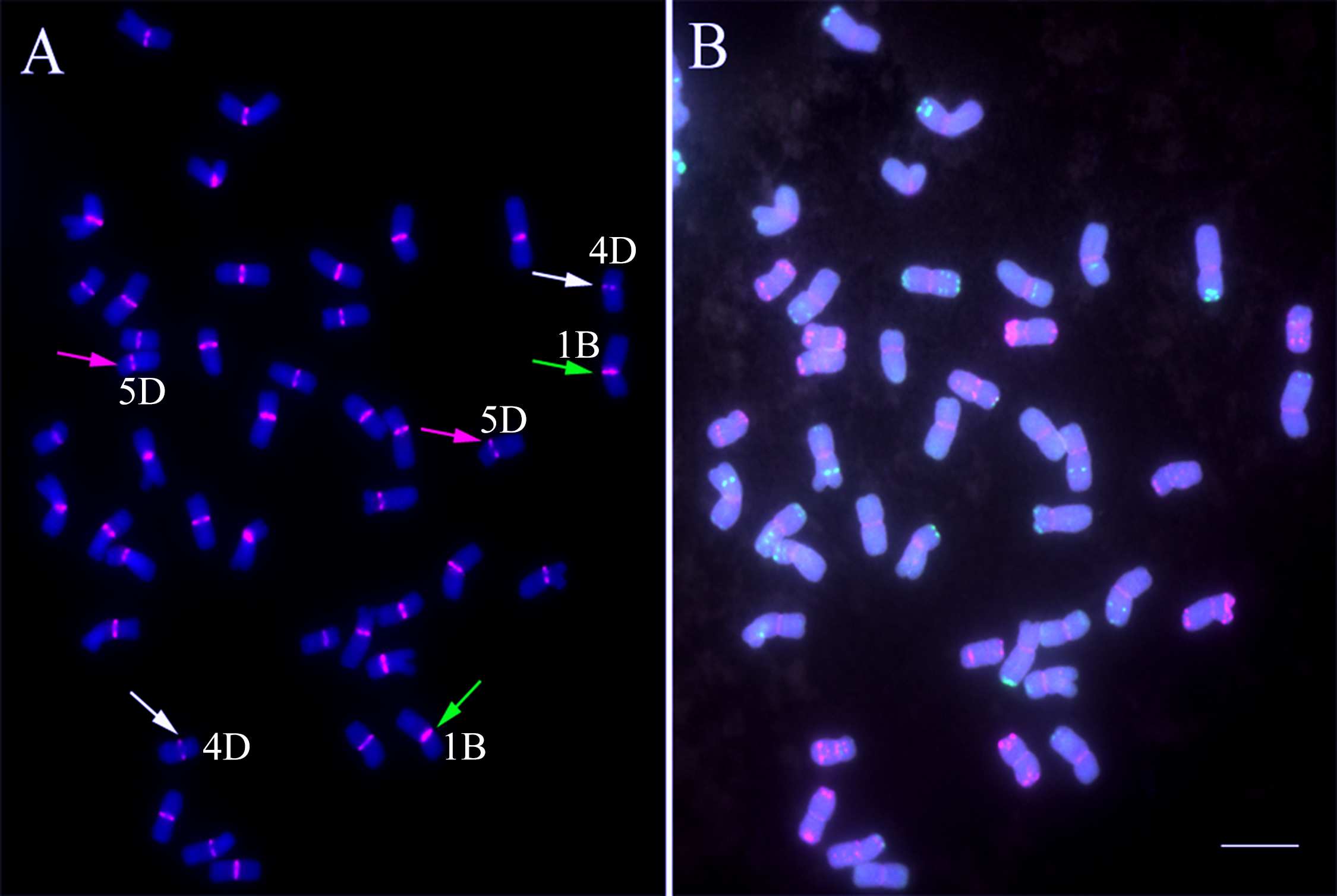

Supplement: S1 Fig — (A) FISH of the CS chromosomes with CRW (red). DAPI staining is labeled in blue. (B) Karyotype analysis of CS using the pAsI (green) and pSc119.2 (red) probes. DAPI staining is labeled in blue. Bar = 10 μm. The 1B, 4D and 5D chromosomes are indicated by green, white, and red arrows, respectively. (TIF) [file pgen.1005997.s001.tif]

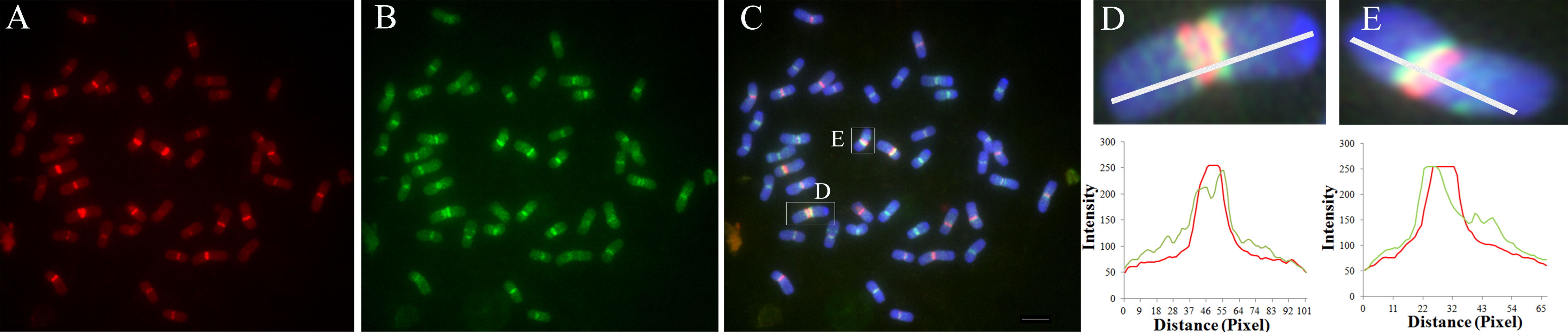

Supplement: S2 Fig — (A)-(C) FISH of TAI-14-2 on chromosomes in Th. intermedium. The CRW sequences are labeled in red, TAI-14-2 is labeled in green, and DAPI staining is labeled in blue. Bar = 10 μm. (D)-(E) Line profile plots of the distributions of the intensity of the CRW and TAI-14-2 signals along the white line of selected chromosomes from (C). (TIF) [file pgen.1005997.s002.tif]

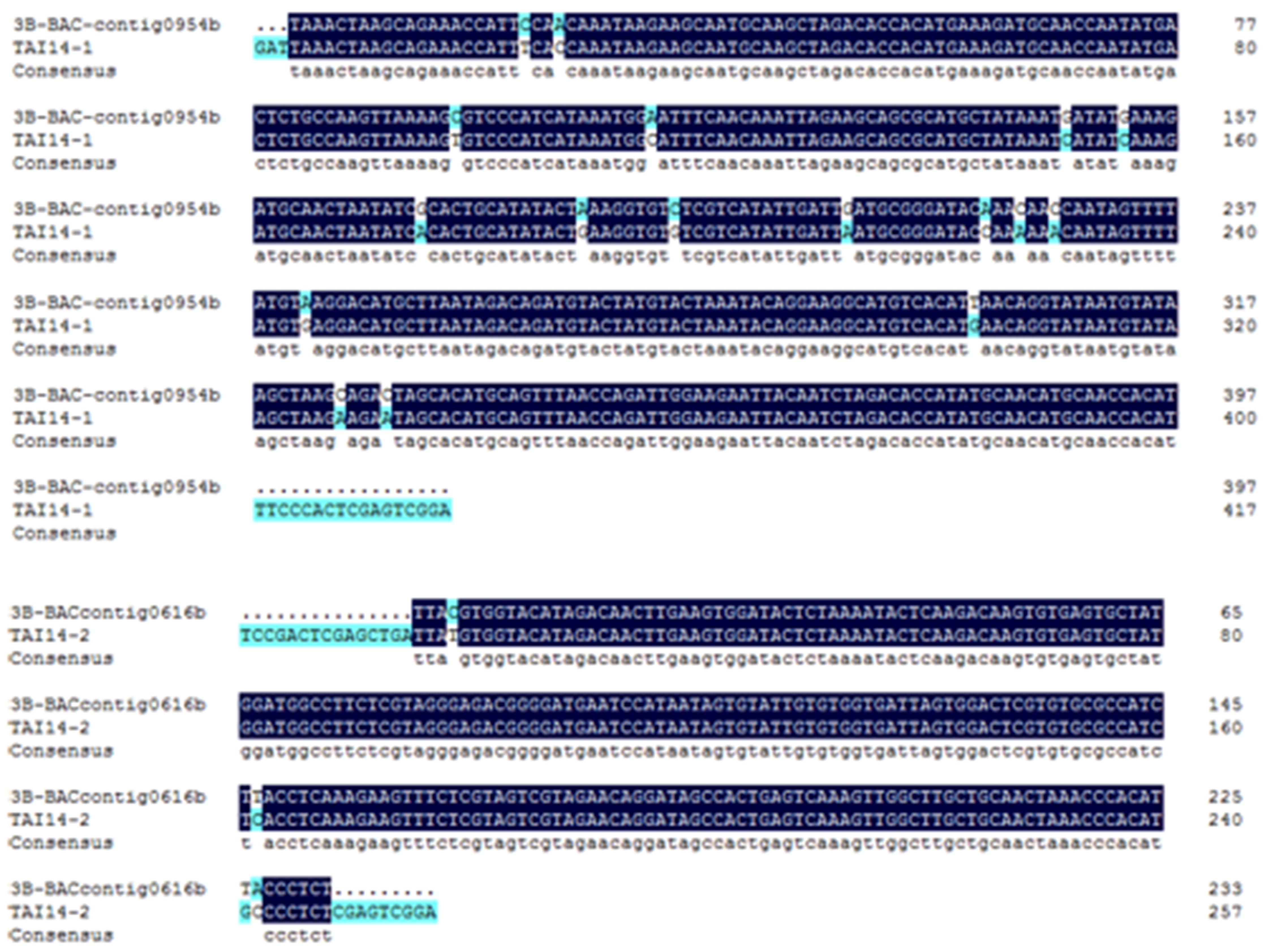

Supplement: S3 Fig — (TIF) [file pgen.1005997.s003.tif]

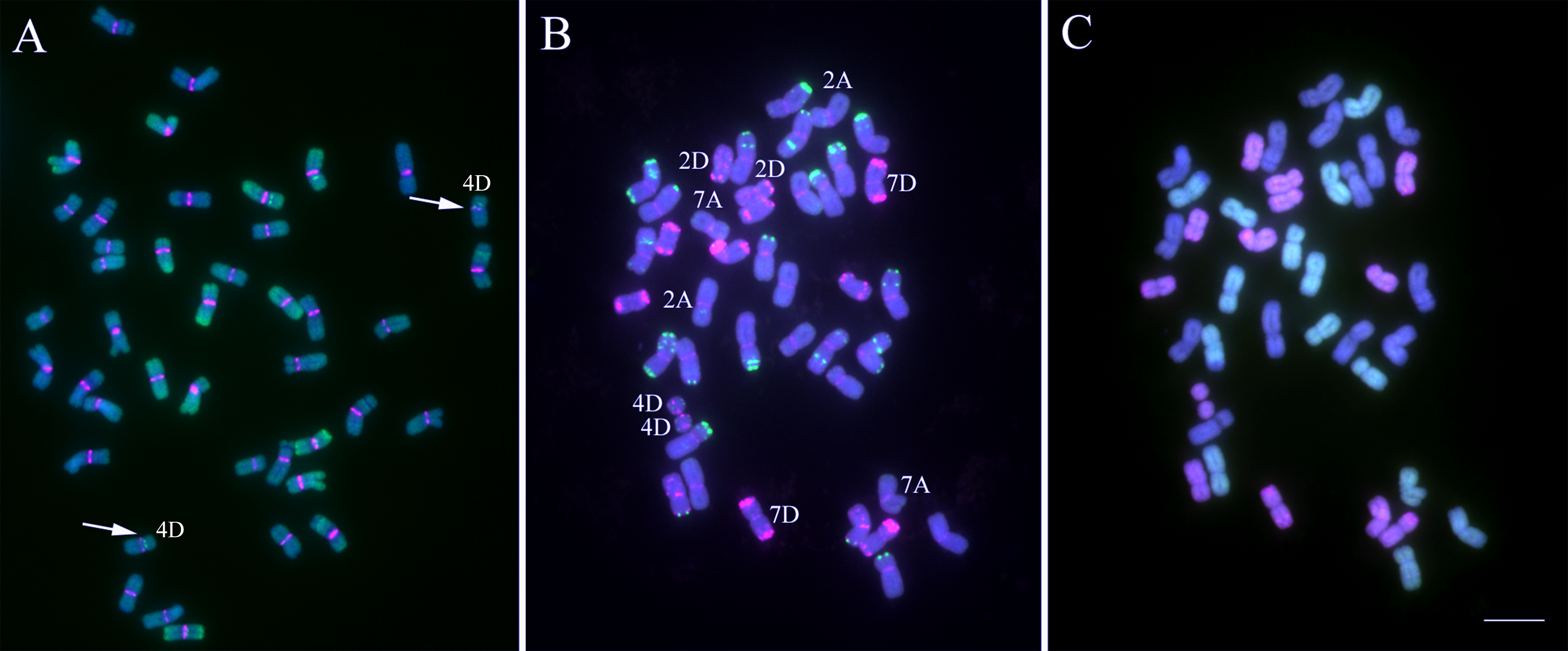

Supplement: S4 Fig — (A) FISH of 4DS-1 on chromosomes in Chinese Spring. The CRW sequences are labeled in red, 4DS-1 is labeled in green, and DAPI staining is labeled in blue. The arrows indicate the 4D chromosomes. (B) Karyotype analysis of 4DS-1 in 4DS using the pAsI (green) and pSc119.2 (red) probes. DAPI staining is labeled in blue. The 2A, 7A, 2D, 4D and 7D chromosomes are indicated in the figure. (C) Multi-color GISH of line 4DS. The DNA for the wheat A genome is labeled in green, the DNA for the D genome is labeled in red, and the DNA for the B genome is used as a block. Bar = 10 μm. (TIF) [file pgen.1005997.s004.tif]

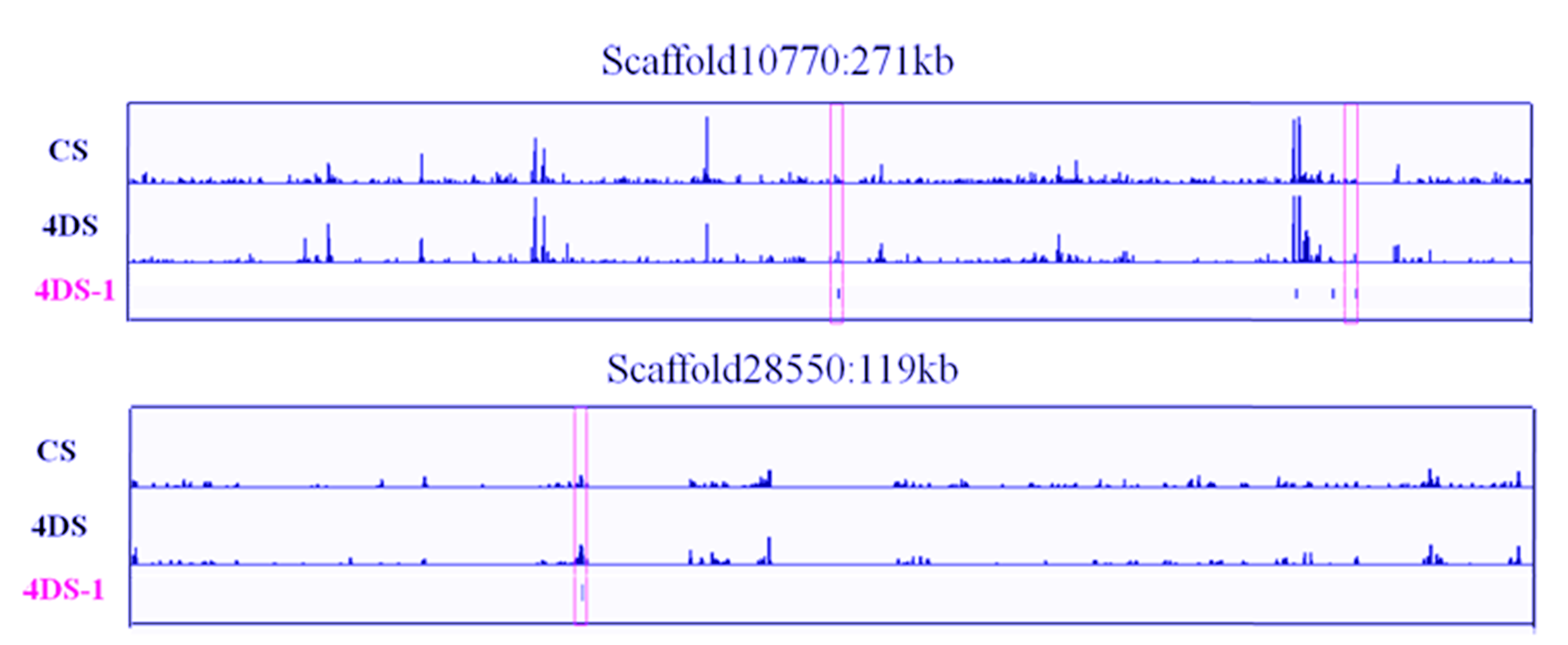

Supplement: S5 Fig — The novel centromeric sequence 4DS-1 shows mapping differences between CS and 4DS in Scaffolds10770 and 28550, as indicated by the magenta boxes. (TIF) [file pgen.1005997.s005.tif]

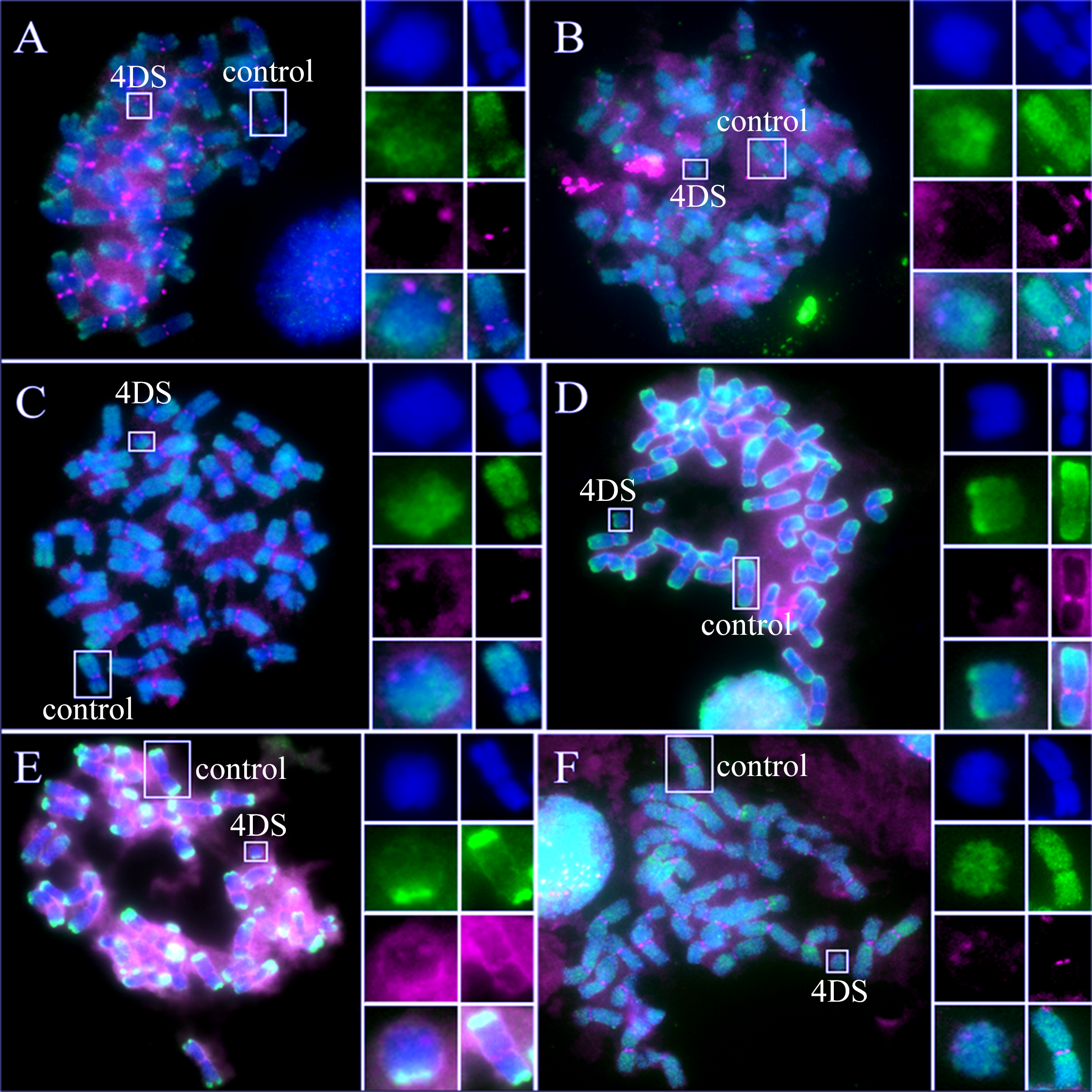

Supplement: S6 Fig — The immunostaining with antibodies against (A) H2AZ; (B) H3K4me2; (C) H3K4me3; (D) H3K27me2; (E) H3K27me3; and (F) H3K9me2 –labeled in green. CENH3 is labeled in red. Bar = 10 μm. (TIF) [file pgen.1005997.s006.tif]

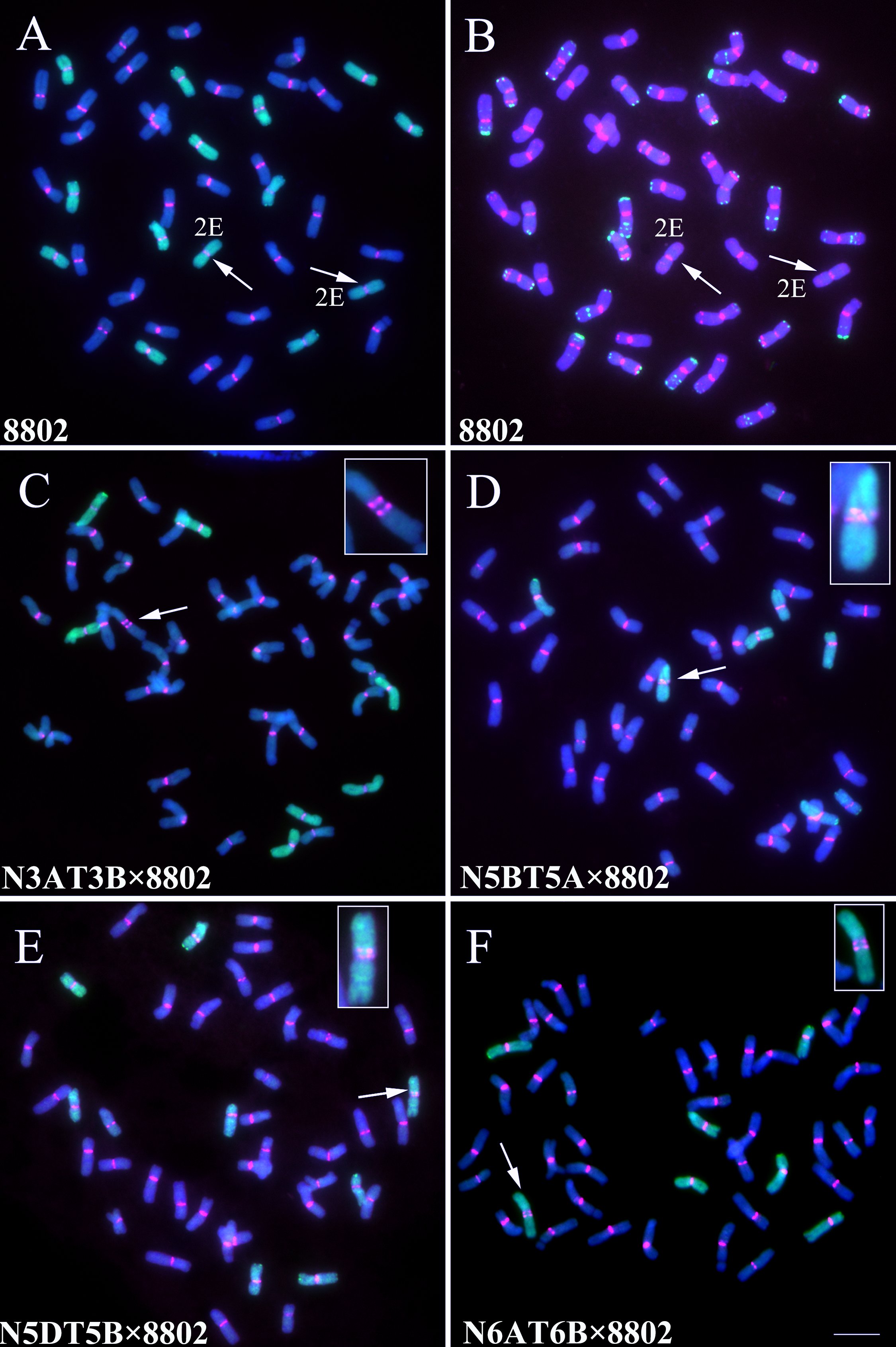

Supplement: S7 Fig — (A) Chromosomes of 8802. The genomic DNA of Th. elongatum is labeled in green, the CRW sequences are labeled in red, and DAPI staining is labeled in blue. (B) Karyotype analysis of 8802. pAsI is labeled in green, and pSc119.2 is labeled in red. The arrows indicate the 2E chromosome. (C)-(F) FISH analysis of the F1 hybrids of N3AT3B×8802 (C), N5BT5A×8802 (D), N5DT5B×8802 (E) and N6AT6B×8802 (F). The insets show high-magnification images of the chromosomes with two centromeric regions. The arrows indicate the chromosomes with changes in their centromeres. Bar = 10 μm. (TIF) [file pgen.1005997.s007.tif]

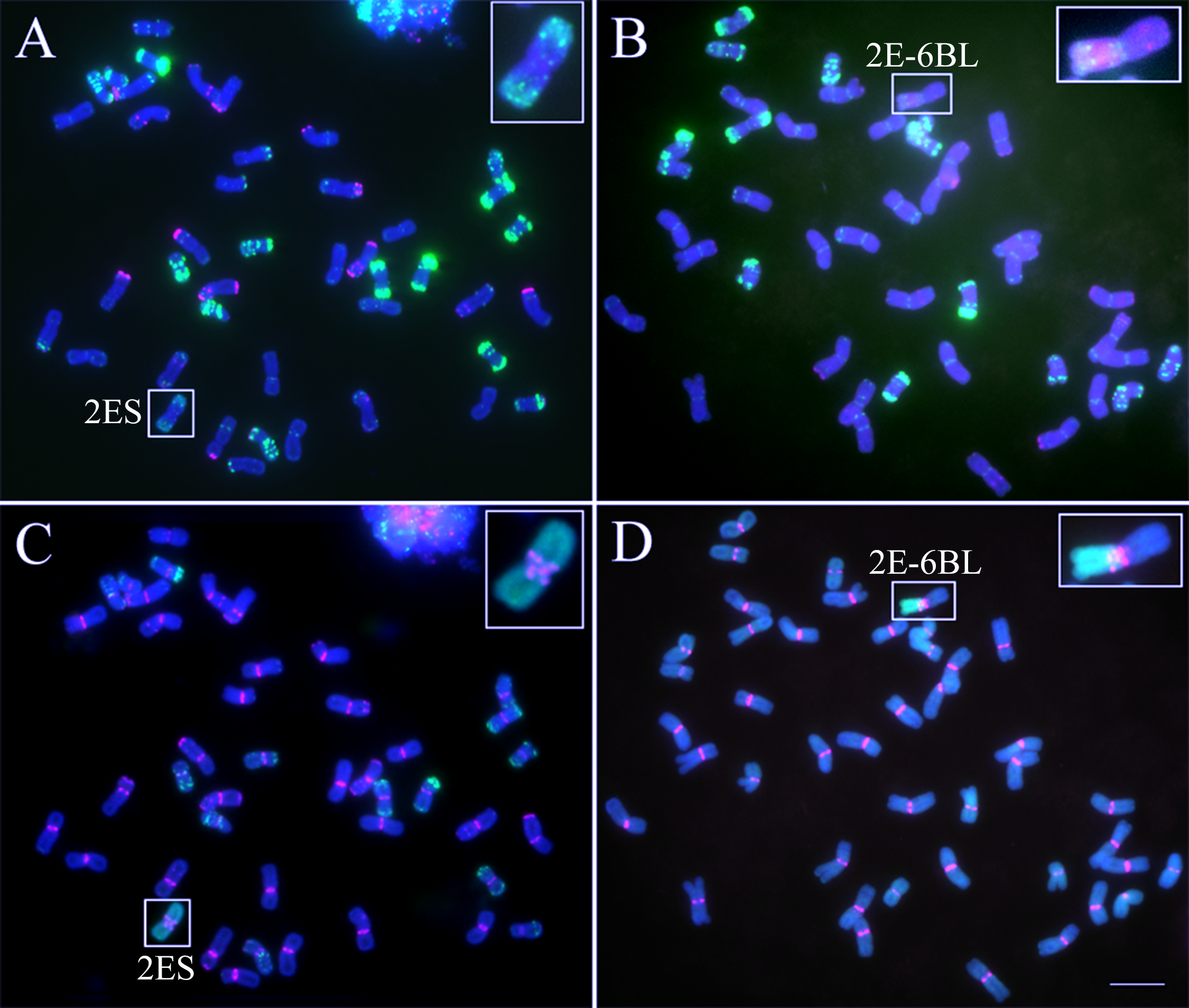

Supplement: S8 Fig — (A) and (B). Karyotype analysis of two different progeny of the three-locus centromeres using the pAsI (green) and pSc119.2 (red) probes. (C) and (D). FISH and GISH on two different progeny with chromosomes bearing a three-locus centromere. The genomic DNA of Th. elongatum is labeled in green, the CRW sequences are labeled in red, and DAPI staining is labeled in blue. The insets show high-magnification images of the chromosomes with three-locus centromeres. Bar = 10 μm. (TIF) [file pgen.1005997.s008.tif]

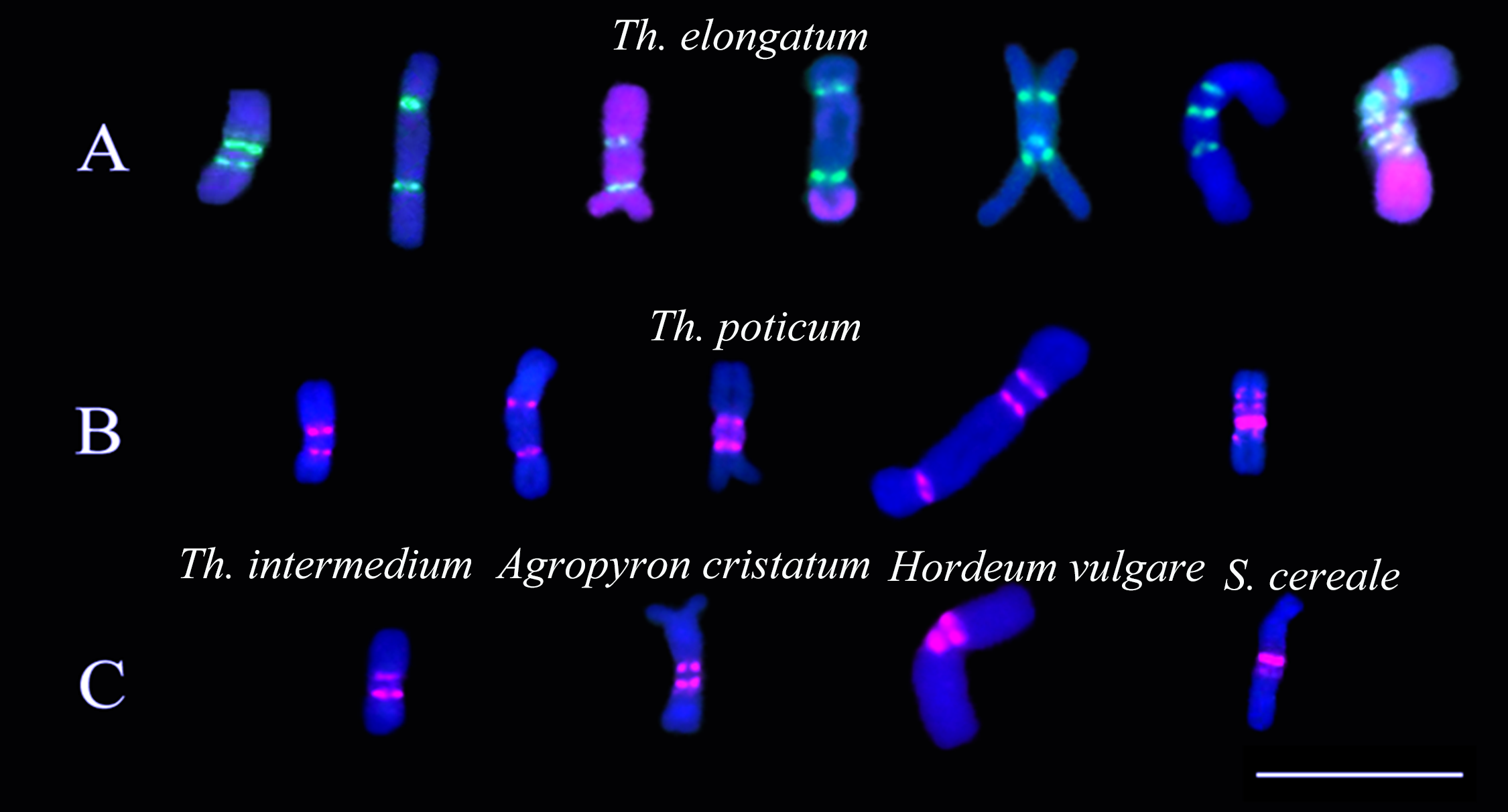

Supplement: S9 Fig — (A) FISH and GISH of the dicentric and multi-centric chromosomes in hybrids of wheat and Th. elongatum. The DNA of Th. elongatum is labeled in red, the CRW sequences are labeled in green, and DAPI staining is labeled in blue. In (B) and (C), the CRW sequences are labeled in red. (B) FISH of the dicentric and multi-centric chromosomes in hybrids of wheat and Th. poticum. (C) FISH of the dicentric chromosomes in hybrids of wheat and Th. intermedium, Agropyron cristatum, Hordeum vulgare and S. cereale. Bar = 10 μm. (TIF) [file pgen.1005997.s009.tif]

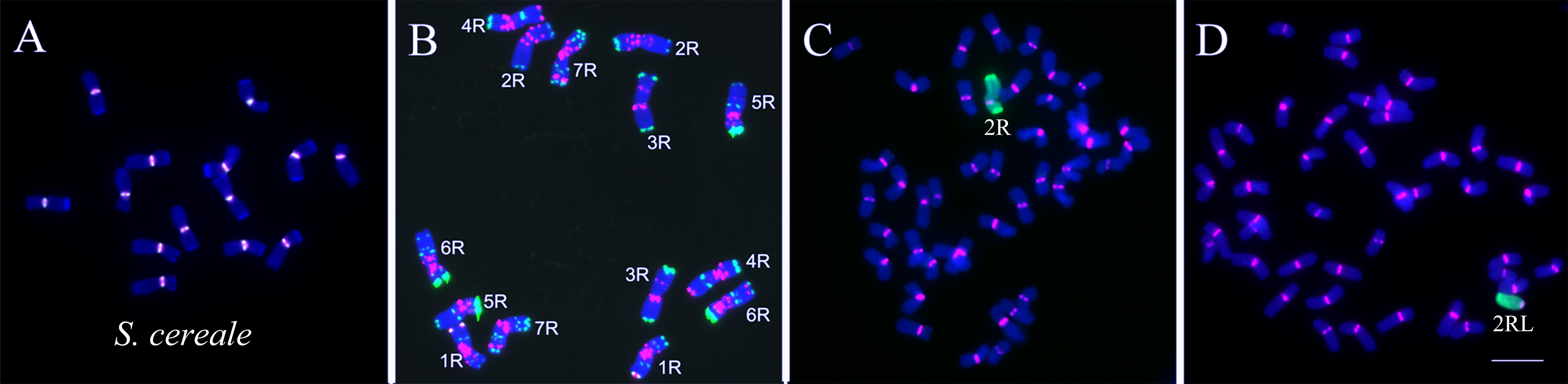

Supplement: S10 Fig — (A) FISH of S. cereale Kustro with the CRW (red) and pAWRC.1 (green) probes. (B) Karyotype analysis of S. cereale Kustro using the AAC (red) and pSc119.2 (green) probes. (C) and (D) FISH analysis of the 2R and 2RL addition lines. The genomic DNA of rye is labeled in green, the CRW sequences are labeled in red, and DAPI staining is labeled in blue. Bar = 10 μm. (TIF) [file pgen.1005997.s010.tif]

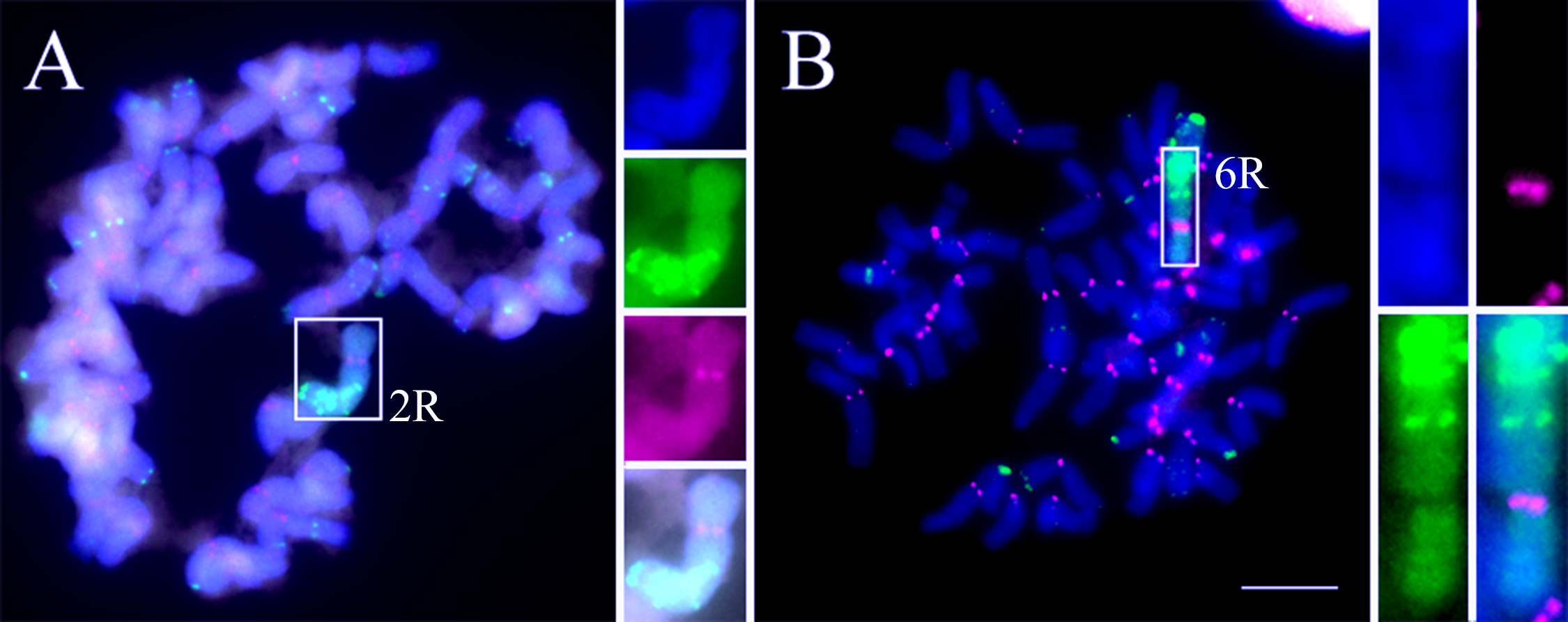

Supplement: S11 Fig — (A) and (B). The novel 2R and 6R addition lines, respectively. The genomic DNA of rye is labeled in green, CENH3 is labeled in red, and DAPI staining is labeled in blue. The insets show high-magnification images of the chromosomes with expanded centromeres. Bar = 10 μm. (TIF) [file pgen.1005997.s011.tif]
