# Supplementary material for: De Novo Centromere Formation and Centromeric Sequence Expansion in Wheat and its Wide Hybrids
Source: PLoS Genet. 2016 Apr 25;12(4):e1005997. doi: 10.1371/journal.pgen.1005997 (PMC4844185; doi:10.1371/journal.pgen.1005997)
Supplement: S1 Table — (DOCX) [file pgen.1005997.s012.docx]

**Table S1. Statistics of the ChIP-Seq Mapping Results.**

| Samples | Total reads | | Total mapped reads | | Nonredundant and mappable reads | |
| --- | --- | --- | --- | --- | --- | --- |
|  | Reads | Percentages | Reads | Percentages | Reads | Percentages |
| Mapped with *Ae. tauschii* (D) genome sequence | | | | | | |
| 4DS | 61749280 | 100% | 30301196 | 49.07% | 28416762 | 46.02% |
| CS | 64132612 | 100% | 30639866 | 47.68% | 28315110 | 44.15% |
| Mapped with *T. aestivum* Chinese Spring (AABBDD) genome sequence | | | | | | |
| 4DS | 61749280 | 100% | 42676651 | 69.11% | 40300845 | 65.27% |
| CS | 64132612 | 100% | 43160672 | 67.30% | 40337046 | 62.90% |
